# The Stability of a Mixed-Phase Barium Cerium Iron Oxide under Reducing Conditions in the Presence of Hydrogen

**DOI:** 10.3390/molecules28031429

**Published:** 2023-02-02

**Authors:** Benjamin Rosen, Karl Sohlberg

**Affiliations:** Chemistry Department, College of Arts and Sciences, Drexel University, Philadelphia, PA 19104, USA

**Keywords:** hydrogen, stability, DFT, membrane, perovskite oxide, separation

## Abstract

Metal oxide perovskite materials show promise for use as hydrogen separation membranes, but metal oxides can dehydrate in the presence of hydrogen to the point of decomposition. The stability of a material in the presence of hydrogen is necessary for an effective hydrogen separation membrane. The stability of a mixed phase metal oxide perovskite (BaCe_0.85_Fe_0.15_O_3-δ_-BaCe_0.15_Fe_0.85_O_3-δ_) was investigated using first-principles thermodynamics calculations based on density functional theory to examine the possible reduction processes on the surface of the material. It was found that for either phase of the material, the loss of H_2_ becomes thermodynamically favorable over the formation of oxygen vacancies once oxygen vacancy defects exist on the surface. Additionally, both phases of the material become more stable with respect to the dehydration or loss of oxygen with increasing concentrations of surface oxygen vacancies. Under the conditions of commercial hydrogen production (~400–1100 K), it is more thermodynamically favorable for H_2_ to desorb from the BaCe_0.85_Fe_0.15_O_3-δ_ phase. Examination of the atomic-scale structure indicates that the degree of coordination of surface metal atoms in this material may control the stability of the material in reducing environments.

## 1. Introduction

As humanity moves away from fossil fuels as a primary energy source, hydrogen is widely seen as a clean alternative energy carrier. Consequently, hydrogen demand is predicted to outpace production over the next decade [1,2]. A fundamental bottleneck in current commercial methods of hydrogen production, steam reforming or dehydrogenation of hydrocarbons, is the energy- and time-intensive process of hydrogen isolation from the resulting product mixtures [3]. Membrane reactor systems are an attractive solution to remediate this process bottleneck. Membrane reactors have not yet seen widespread commercial adoption because current metallic membrane materials are prohibitively costly and have short cycle lives [4,5]. Metal oxide ceramics that can function as hydrogen separation membranes are a class of materials that have the potential to address the issues with current commercially viable hydrogen separation membranes [6,7]. Many metal oxide materials decompose via dehydration in hydrogen rich environments—a consequence of reduction by hydrogen resulting in the dehydration of the material [8,9]. For a material to properly function as a hydrogen separation membrane, the material must be stable with respect to both reduction and oxidation in hydrogen-rich environments so that hydrogen gas can repeatedly adsorb at the retentate surface and desorb from the permeate surface without damaging the membrane. Additionally, for hydrogen gas to be collected from the permeate surface of a hydrogen separation membrane, desorption of hydrogen gas must be thermochemically preferred over dehydration. Dehydration here only refers to the evolution of water as a product, not the loss of absorbed water. Structurally stable materials are paramount for the development and manufacture of commercial hydrogen separation membranes. In order to design improved ceramic oxide hydrogen separation membranes, it is necessary to ensure the materials will be stable in the harsh chemical environments of commercial hydrogen production.

Some metal oxide perovskite materials appear to be stable under desorption of hydrogen over a wide temperature range, and exhibit hydrogen flux rates comparable to metal membranes [6,10]. Stability combined with unique optical, electronic, and catalytic properties gives materials with the perovskite structure wide-ranging applications [11]. Cheng et al. [10] reported an investigation of a ternary metal oxide ceramic as a hydrogen separation membrane material. The material investigated by Cheng et al. [10] was reported to be a mixed-phase material composed of two complimentary defect perovskite structures, BaCe_0.85_Fe_0.15_O_3-δ_ and BaCe_0.15_Fe_0.85_O_3-δ_ (BCF8515 and BCF1585, respectively), in a 50/50 stoichiometric ratio. Cheng et al. [10] did not report the detection of water in the permeate gas sweep. Only H_2_, N_2_ bleed over from the retentate side of the membrane, and the argon permeate sweep gas were reported [10]. Similar results were reported by Xia et al. [12]. Many metal oxides reduce and desorb water in the presence of H_2_ due to the thermodynamic stability of water [8,13,14]. The apparent lack of dehydration at the permeate surface upon reduction by H_2_ indicates that BCF8515-BCF1585 may be stable with respect to reduction in hydrogen atmospheres. Stability in the presence of hydrogen is necessary for commercially viable materials. Understanding what material properties lead to stability in reducing hydrogen atmospheres is fundamental to designing improved hydrogen separation membrane materials.

A theoretical investigation of the stability of BCF8515-BCF1585 to reduction at the permeate surface was performed. To do so, the thermodynamics of several possible competing and sequential reactions at the surface of BCF8515 and BCF1585 crystals were determined from first principles with density functional theory (DFT) calculations, the results and interpretation of which are reported here.

## 2. Methods

### 2.1. Computational Details

#### 2.1.1. Software Parameters

All electronic structure calculations were performed with density functional theory using the PW91 [15] approximation to the exchange-correlation and were carried out with the Vienna ab initio Simulation Package (VASP) [16,17,18]. All calculations employed a plane-wave basis subject to a cutoff energy of 400 eV. The pseudopotentials used were all the standard VASP-provided projector augmented wave (PAW) pseudopotentials for use with general gradient approximation (GGA) exchange-correlation functionals [19]. All geometry optimizations were performed using either the conjugate gradient relaxation algorithm, or the RMM-DIIS relaxation algorithm [16]. The RMM-DIIS algorithm was only utilized in cases where the conjugate gradient algorithm resulted in a bracketing error due to the nuclear geometry being very near a minimum energy configuration. All structural relaxations were terminated when the change in the forces was less than 0.02 $\frac{\mathrm{eV}}{Å\;}$. The k-point sampling density for all calculations was determined by VASP’s built-in automatic k-point grid selector. All k-point grids utilized can be found in S1–S4 of the Supplementary Materials. Gaussian smearing with a smearing width of 0.2 eV was used for all calculations. The 0K total electronic energy values were utilized for all free energy calculations in the standard approximation [20,21].

#### 2.1.2. Structural Models

The initial bulk unit cell structures for all three materials investigated were built by modifying an undoped perovskite material (BaCeO_3_ or BaFeO_3_) having the desired symmetry (cubic or orthorhombic). A supercell of the undoped material was built, such that a B-site metal cation could be substituted for a dopant atom to yield the desired stoichiometry. All bulk structures can be found in S1 of the SI. All *dopant* defects were substitutional only. All structures had 1 dopant atom per 7 atoms of the element being substituted. This yielded slab stoichiometries of Ba_16_Ce_14_Fe_2_O_48_ (to represent BCF8515) and Ba_16_Ce_2_Fe_14_O_48_ (to represent BCF1585) for materials containing no oxygen vacancy defects. Oxygen vacancy defects were added to the surface of each material sequentially by removing a single oxygen atom per surface supercell at each stage of decomposition. All oxygen vacancies were added on the surface of the respective slab models. The ratios of Ce to Fe used were selected to achieve the closest ratio to 15% dopant (15% by atom count, not mass percent) with unit cells sufficiently small to remain computationally tractable. For all structures investigated, all dopant defects were located within the bulk of the material or on the bottom exposed surface, not the surface interacting with adsorbed species and containing oxygen defects.

The preferred surface exposure for each material investigated was determined by finding the surface structure that minimized the surface energy. Each surface energy calculation was obtained by performing a geometry relaxation on a slab with the desired Miller index surface exposed and calculating the surface energy by

(1) $$\mathrm{Surface}\;\mathrm{Energy}=\frac{E_{\mathrm{slab}}-{n*E}_{\mathrm{bulk}}}{2*A_{\mathrm{slab}}}$$

where $E_{\mathrm{slab}}$ is the total energy of the slab, $E_{\mathrm{bulk}}$ is the total energy of a unit cell of the bulk material, n is a scaling factor to adjust the total energy of the bulk to the number of unit cell equivalents in the slab, and $A_{\mathrm{slab}}$ is the surface area of the exposed surface. Every slab utilized for surface energy calculations was cleaved from 2 bulk unit cells (n=2). The bottom two layers of atoms were frozen for each surface energy calculation to simulate the bulk material that exists between two exposed surfaces. In asymmetric cases, a dipole correction was applied along the vector normal to the exposed surface. For all slab calculations symmetric slabs were utilized wherever possible. The surface energies were calculated for the lowest and second-lowest order Miller index surfaces for each material. The lowest energy surfaces identified for each material were (010) surface for cubic BCF8515, (101) for cubic BCF1585, and (011) surface for orthorhombic BCF8515. All stability calculations were performed on the respective lowest energy surface for each material. All slab structures can be found in S2–S4 of the SI. Zhu et al. [13] reported that the structure of BCF1585 remains in the cubic space group for temperatures below 1200 °C. Since the cubic structure of BCF1585 presented by Cheng et al. [10] is unambiguous, no investigation of an orthorhombic phase of BCF1585 was performed.

#### 2.1.3. Selection of Crystal Structures

Selection of the crystal structure for BCF1585 was accomplished by comparing the crystal structure presented by Cheng et al. [10] with the common cubic space groups of BaFeO_3_. BCF1585 was identified as being derived from BaFeO_3_ in the cubic *pm*$\bar{3}$*m* space group.

For the BCF8515 phase, Zhu et al. [13], Cheng et al. [10], and Xia et al. [12] all reported an orthorhombic structure; however, none reported a possible space group. Cheng et al. [10] reported BCF8515 as having an orthorhombic symmetry that results from an elongation of one axis from the BaCeO_3_ *pm*$\bar{3}$*m* cubic structure. For BaCeO_3_, elongation of one axis results in an orthorhombic structure having symmetry in the *pnma* space group. Cheng et al. [10] reported that both phases (Fe or Ce dominant) form structures with the same symmetry as the corresponding undoped materials (BaFeO_3_ and BaCeO_3_, respectively). As per Knight [22], BaCeO_3_ undergoes 3 temperature-dependent phase transitions. According to Knight [22], BaCeO_3_ transitions to the cubic *pm*$\bar{3}$*m* space group at 900 °C. Cheng et al. [10] reported calcining both BCF8515-BCF1585 samples at 1370 °C. The subsequent hydrogen permeation testing was performed from 850 to 950 °C. The temperatures at which the BCF8515–BCF1585 samples were treated and tested were high enough to facilitate a transformation to the cubic phase, assuming BCF8515 behaves similarly to BaCeO_3_.

**Figure 1:**
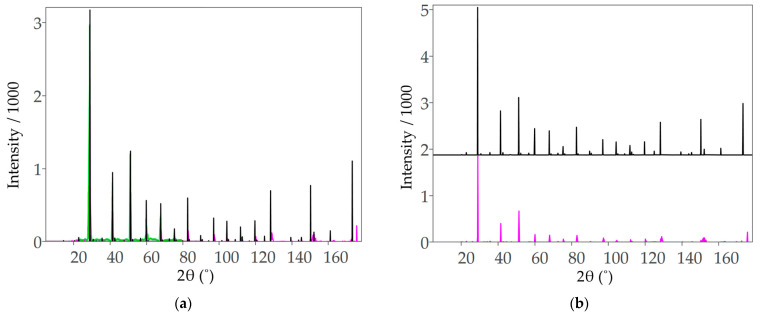
(**a**) Simulated X-ray diffraction patterns for orthorhombic (pink) and cubic (black) BCF8515 for 2θ from 0° to 180°, overlayed with data extracted from Zhu et al. [13] (green). (**b**) Simulated X-ray diffraction patterns for orthorhombic and cubic BCF8515 for 2θ from 0° to 180°, stacked. Simulated data generated using CrystalDiffract^®^, CrystalMaker Software Ltd. (Oxford, UK), (www.crystalmaker.com, accessed on 26 December 2022).

In an effort to determine the most probable structure of BCF8515, three possible structures were compared: two orthorhombic structures derived by doping the *pnma* phase of BaCeO_3_ with an iron defect (Fe substitution at each of the two possible Ce sites) and a cubic phase derived from the *pm*$\bar{3}$*m* phase of BaCeO_3_. Each bulk structure was relaxed as per the method described in Section 2.1.1 and Section 2.1.2 for unit cells of the same stoichiometry. The lower energy of the two orthorhombic phases was identified from total energy calculations and selected for stability investigation. The cubic structure had the highest total energy. In an attempt to resolve the ambiguity in the results presented by Cheng et al. [10], and the contradiction with the preferred phase under the reaction conditions, based on the results of Knight [22], the predicted 2θ plots for the orthorhombic and cubic structures were compared. The calculated 2θ plots can be found in Figure 1. From the 2θ plots, it can be seen that the only noticeable peak shifts that occur between the two structures can be found at 2θ > 80°. The orthorhombic structure does have some peak broadening compared to the cubic structure; however, this feature is not pronounced until 2θ > 80°. Since the ambiguity between the lowest energy structure and the structure likely to exist under the reaction conditions could not be resolved, both the orthorhombic and cubic phases of BCF8515 were investigated, and the results for both are presented herein.

### 2.2. Stability Study Model

#### 2.2.1. Protonation: Decomposition or Desorption

The goal of this study was to examine the thermodynamics of forming reduction products from the protonated surface of barium cerium iron oxides to investigate the stability of these materials in the chemical environment of a hydrogen-generating membrane reactor. Three possible reactions yielding gas-phase products were considered: 1. The desorption of H_2_. 2. The desorption of H_2_O and the formation of an oxygen vacancy. 3. The spontaneous formation of an oxygen vacancy. These reactions are represented by reactions (I)–(III) below, where S denotes the slab model and $V^{O}$ an oxygen vacancy.

$$2H/S\to H_{2}+S\;(I)\;2H/S\to H_{2}O+V^{O}/S\;(\mathrm{II})\;2H/S\to \frac{1}{2}O_{2}+2{\mathrm{HV}}^{O}/S\;(\mathrm{III})$$

The enthalpy and entropy changes were calculated for each of the reactions (I)–(III) and were used to predict the temperature at which the Gibbs free energy for each process becomes negative (passes equilibrium to become spontaneous). This was repeated for surfaces with an increasing number of oxygen vacancies for each material. The thermodynamically favored processes for each possible reaction on each possible surface were determined by calculating the temperature-dependent Gibbs free energy change for each reaction and comparing across the reactions to determine the thermodynamically preferential reaction on each surface.

#### 2.2.2. Enthalpy Change Calculations

The total change in enthalpy for each representative reaction was calculated by first performing an electronic total energy calculation on each reactant and product. The change in enthalpy was calculated as

(2) ΔEnthalpy=Σ total energy products−Σ total energy reactants

since PV work is negligible for the systems of interest due to the low pressure (380 pascal) of products at the surface. The total energy calculations for each gas-phase molecule (H_2_, O_2_, H_2_O) were performed by placing a single molecule in the center of a 10 × 10 × 10 Å unit cell and performing a full geometry optimization of the molecule. For all calculations that utilize ½ O_2_, the total enthalpy of O_2_ was multiplied by a factor of ½.

#### 2.2.3. Entropy Change Calculations

Calculation of the change in entropy from reactants to products in the representative reactions began with the following assumptions: All the “reactant” species for all the representative reactions were bound to periodic solids, so there could only be vibrational degrees of freedom (movement of atoms in the desorbing species relative to the surface slab, i.e., no rotation or translation). The products in the representative reactions included gas-phase species, which have vibrational, rotational, and translational degrees of freedom. All reactions were assumed to be in the electronic ground state, so electronic degrees of freedom were not considered in the canonical ensemble. The change in entropy was taken as the entropy difference between the vibrational degrees of freedom for the atoms constituting the gas-phase species when bound to the solid surface and the total entropy of the gas-phase species. The entropy change of the remainder of the surface was neglected because it was assumed to be an insignificant change in relation to the entropy change due to the formation of product gas [23]. This yielded a change in entropy for the formation of a gas-phase molecule from the surface of interest.

The absolute entropies were calculated in the standard way,

(3) $$S=k_{B}\ln\left(Q\right)$$

where $k_{B}$ is Boltzmann’s constant and Q is the canonical partition function representing all the accessible states. For the gas-phase species, the canonical partition function was taken as the product of the $q_{\mathrm{rot}}$ and $q_{\mathrm{vib}}$ (rotation and vibration, respectively) partition functions under the rigid rotor and harmonic oscillator approximations,

(4) $$Q_{\mathrm{gas}}=q_{\mathrm{vib}}\times {\;q}_{\mathrm{rot}}$$

since the electronic partition function was assumed to be in the ground state ($q_{\mathrm{electronic}}=1$). The vibrational and rotational partition functions were calculated in the standard way:

(5) $$q_{\mathrm{vib}}=\prod_{i=1}^{n}\frac{\exp\left(-\frac{{h\nu }_{i}}{k_{B}T}\right)}{1-\exp\left(-\frac{{h\nu }_{i}}{k_{B}T}\right)}$$

where $\nu_{i}$ is the i^th^ vibrational frequency, $k_{B}$ is Boltzmann’s constant, h is Planck’s constant, T is the temperature in kelvin, and

(6) $$q_{\mathrm{rot}}=\frac{\pi^{\frac{1}{2}}}{\sigma }\prod_{i=1}^{n}{\left(\frac{8\pi^{2}I_{i}k_{B}T}{h^{2}}\right)}^{\frac{1}{2}}$$

where $I_{i}$ is the $i^{\mathrm{th}}$ rotational moment of inertia, $k_{B}$ is Boltzmann’s constant, h is Planck’s constant, σ is a factor correcting for degenerate rotations, and T is the temperature in kelvin. All vibrational frequencies were obtained by performing a vibrational eigenvalue calculation restricted to the atoms of interest with VASP. All moments of inertia were calculated from the equilibrium nuclear coordinates as calculated with VASP. The translational entropy was approximated using the Sakur–Tetrode equation for translational entropy [24], and was added to the total entropy of the gas-phase species, in the same manner as Cai and Sohlberg [25].

(7) $$S_{\mathrm{trans}}=k_{B}\cdot \left(\ln\left(\frac{k_{B}T}{P}\right)+\frac{3}{2}\cdot \ln\left(\frac{2{\pi \mathrm{mk}}_{B}T}{h^{2}}\right)+\frac{5}{2}\right)$$

where $k_{B}$ is Boltzmann’s constant, T is the temperature in Kelvin, P is the partial pressure of the gas in pascals, and m is the mass of the gas.

For the solid-phase reactants, Q was taken as just the vibrational partition function for the atoms that become gas-phase species in the specific reaction under investigation. The total entropy change was calculated as the difference in the entropy of the reactants and products:

(8) $${\Delta S}_{\mathrm{total}}=k_{B}\ln\left(\frac{Q_{\mathrm{gas}}}{Q_{\mathrm{solid}}}\right)+S_{\mathrm{trans}}$$

where $Q_{\mathrm{gas}}$ is the canonical partition function for the product gas, $Q_{\mathrm{solid}}$ is the partition function for the reactants on the surface.

The entropy change for each reaction was calculated at a pressure of 380 pascal, the permeate pressure as reported by Cheng et al. [10], while allowing temperature to remain an independent variable. The temperature-dependent entropy change was substituted into the equation for the Gibbs free energy. The temperature-dependent Gibbs free energy change for each reaction was plotted on the same set of axes for each respective surface and compared. From the changes in Gibbs free energy, the most thermodynamically favorable reactions were identified for each surface. The process was continued stepwise by sequentially increasing numbers of oxygen vacancies until the free energy change for the formation of an oxygen vacancy was greater than that for the desorption of H_2_ over all relevant temperatures.

## 3. Results

### 3.1. Results of Free Energy Change Analysis

The method prescribed in Section 2 (Methods) was applied to the three structures of interest: orthorhombic BCF8515, cubic BCF8515, and cubic BCF1585. Reactions (I)–(III) (see Section 2.2.1) were modeled on the lowest energy surface for each respective material, and each relevant oxygen-vacancy-containing derivative structure. The change in free energy was compared across reactions (I)–(III). For each of reactions (I)–(III) from each surface examined, the enthalpy change was found to be positive, demonstrating that all three reactions are endothermic. The entropy change was found to be positive over all temperature ranges for all three reactions considered. Thus, at elevated temperatures, the reactions reach equilibrium (ΔG = 0), and eventually became spontaneous (ΔG < 0). At relevant temperatures, the relative free energy changes were found to be dominated by the enthalpy changes.

#### 3.1.1. Orthorhombic BCF8515

**Figure 2:**
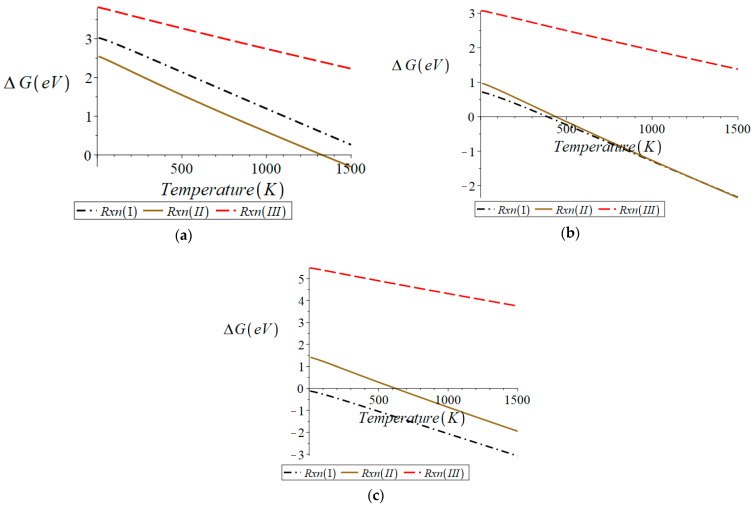
Surface of orthorhombic BCF8515 for (**a**) oxygen-vacancy-free surface, (**b**) surface with 1 oxygen vacancy, (**c**) surface with 2 oxygen vacancies. Plots were generated in Maple^TM^.

Cheng et al. [10] reported that the BCF8515 they synthesized had an orthorhombic structure. The orthorhombic structure utilized in this work was found to be the lowest energy structure of BCF8515. A symmetric slab was cleaved, exposing the (011) surface, as discussed in Section 2.1.2. The free energy change, ΔG, was calculated for reactions (I)–(III) occurring from the (011) surface of orthorhombic BCF8515 with zero, one, and two oxygen vacancy defects, respectively. ΔG vs. temperature for each of these nine cases can be found in Figure 2. All the equations of the lines plotted in Figure 2 can be found in S5 of the SI. 

From Figure 2, it can be seen that starting from the (011) surface containing no oxygen vacancies (“reactant” slab stoichiometry Ba_16_Ce_14_Fe_2_H_2_O_48_), the smallest free energy change over all temperatures is that of reaction (II) (formation of H_2_O_(g)_). Thus, reaction (II) is the most thermodynamically favorable reaction for a surface with no oxygen vacancy defects. Since reaction (II) generates an oxygen vacancy, the free energy changes for reactions (I)–(III) were calculated for a surface containing one oxygen vacancy (“reactant” slab stoichiometry Ba_16_Ce_14_Fe_2_H_2_O_47_). From Figure 2b it can be seen that the formation of H_2_ from the surface with one oxygen vacancy becomes spontaneous (ΔG<0) at the lowest temperature, though at high temperatures the free energy change for reaction (II) crosses that for reaction (I) (approximately 1200 K). For this reason, reactions (I)–(III) were investigated for the surface containing two oxygen vacancies (“reactant” slab stoichiometry Ba_16_Ce_14_Fe_2_H_2_O_46_). From Figure 2c it can be seen that again reaction (I), formation of H_2_, is more thermodynamically favorable than reactions (II) or (III) and is spontaneous over all temperature ranges. These results demonstrate that the surface of orthorhombic BCF8515 becomes stable to reduction once 0.0215 oxygen defects per Å^2^ exist on the surface of the material. (This value and other surface oxygen vacancy threshold concentrations reported here are upper limits. The actual threshold may be lower. Investigating lower vacancy concentrations would involve calculations employing computationally demanding larger supercells).

In summary, these results indicate that if orthorhombic BCF8515 exists with surface oxygen vacancy defects at a concentration exceeding 0.0215 oxygen vacancy defects per Å^2^, the loss of H_2_ from the protonated surface becomes the dominant reduction process. It was found that the spontaneous formation of oxygen vacancies via a loss of ½ O_2_ does not occur, except at physically irrelevant temperatures. This indicates that all oxygen vacancies in the material will form either intrinsically upon crystallization, or via the dehydration of a protonated surface.

#### 3.1.2. Cubic BCF8515

**Figure 3:**
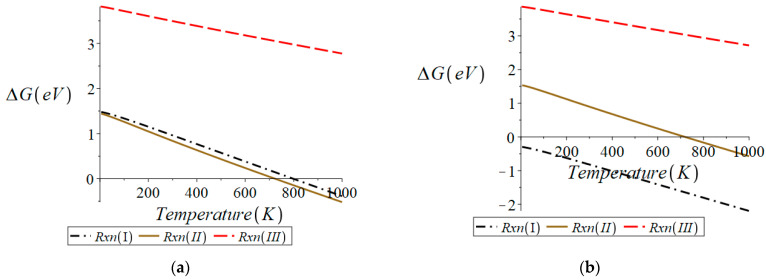
Surface of cubic BCF8515 for (**a**) surface with no oxygen vacancy defects, (**b**) surface with 1 oxygen vacancy defect. Plots were generated in Maple^TM^.

Cubic BCF8515 was examined in the same manner as orthorhombic BCF8515. Figure 3 shows the free energy change of reactions (I)–(III) with respect to temperature for the cubic BCF8515 surface containing no oxygen vacancies and for the surface of cubic BCF8515 containing one oxygen vacancy. All the equations of the lines plotted in Figure 3 can be found in S6 of the SI.

From Figure 3a it can be seen that for the surface containing no oxygen vacancies (“reactant” slab stoichiometry Ba_16_Ce_14_Fe_2_H_2_O_48_), reaction (II) is thermodynamically favored over reactions (I) and (III) over all relevant temperatures. This indicates that the preferred product from the surface containing no oxygen vacancies is H_2_O. Since the preferred product from a surface with no oxygen vacancy defects generates an oxygen vacancy defect, the free energy change for reactions (I)–(III) were investigated for a surface containing a single oxygen vacancy (“reactant” slab stoichiometry Ba_16_Ce_14_Fe_2_H_2_O_47_). From Figure 3b it can be seen that the formation of H_2_ is thermodynamically preferred over reactions (II) and (III) and is spontaneous over all temperatures. This result indicates that the surface of cubic BCF8515 with an oxygen vacancy defect concentration of no greater than 0.0130 vacancies per Å^2^ begins to stabilize to reduction in the presence of hydrogen.

#### 3.1.3. Cubic BCF1585

**Figure 4:**
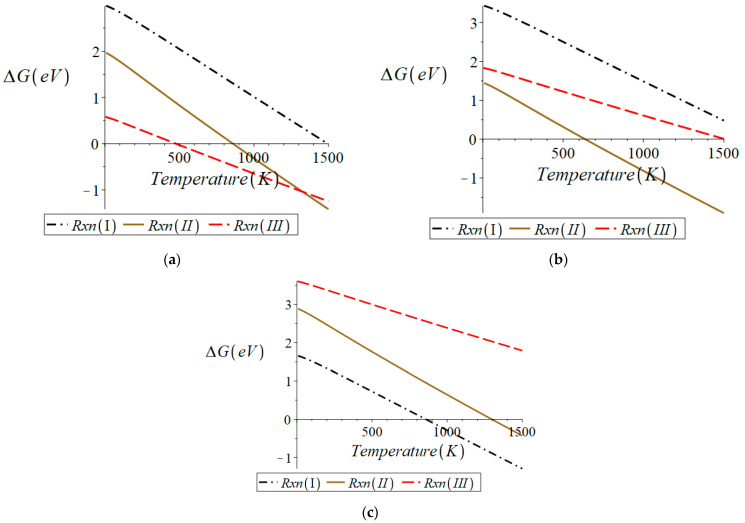
Surface of cubic BCF1585 for (**a**) surface with no oxygen defects, (**b**) surface with 1 oxygen vacancy, (**c**) surface with 2 oxygen vacancies. Plots were generated in Maple^TM^.

Cheng et al. [10] reported that only BCF8515 transports atomic hydrogen through the binary phase material BCF8515-BCF1585. Cheng et al. [10] and Xia et al. [12] also reported that the surface of BCF8515-BCF1585 was composed of a homogeneous mixture of BCF8515 and BCF1585 grains. The stability of cubic BCF1585 was investigated in the same manner as BCF8515. The results obtained can be found in Figure 4. All the equations of the lines plotted in Figure 4 can be found in S7 of the SI.

From Figure 4a, it can be seen that the formation of an oxygen vacancy from the oxygen-defect-free surface (“reactant” slab stoichiometry Ba_16_Fe_14_Ce_2_H_2_O_48_) via the loss of ½ O_2_ is thermodynamically preferred over the other possible reductions, i.e., reactions (I) and (II). At elevated temperatures, reduction via reaction (II) becomes the thermodynamically favored reaction; however, this does not occur until approximately 1350 K, just above the standard operating temperatures of membrane reactors of 1100 K [26,27]. Since the thermodynamically preferred reaction from the oxygen-defect-free surface generates an oxygen vacancy, reactions (I)–(III) were analyzed from a surface containing one oxygen vacancy (“reactant” slab stoichiometry Ba_16_Fe_14_Ce_2_H_2_O_47_). Figure 4b shows the thermodynamically preferred reaction over all temperatures to be reaction (II). From Figure 4c, it can be seen that once a second oxygen vacancy exists on the surface of BCF1585 (“reactant” slab stoichiometry Ba_16_Fe_14_Ce_2_H_2_O_46_), the formation of H_2_ becomes thermodynamically preferred, indicating that the surface is stable to reduction once a surface concentration of at least 0.0222 oxygen vacancies per Å^2^ exist on the surface.

**Figure 5:**
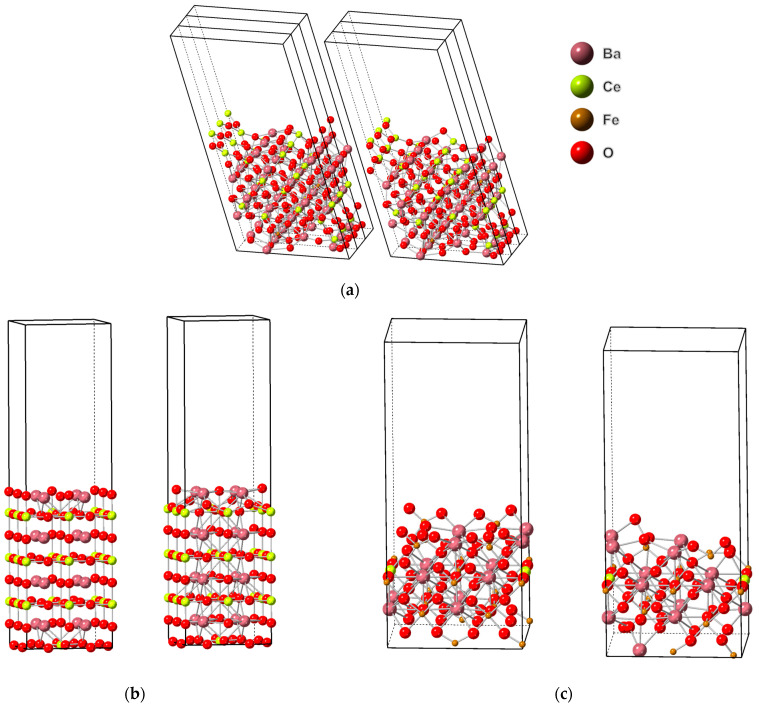
(**a**) Right: orthorhombic BCF8515 with 3 oxygen vacancies on the surface, 3-unit cells in the x-direction. Left: Oxygen-vacancy-defect-free orthorhombic BCF8515 surface, 3-unit cells in the x-direction. (**b**) Right: Cubic BCF8515 with 2 oxygen vacancies on the surface. Left: Oxygen-vacancy-defect-free cubic BCF8515. (**c**) Right: BCF1585 with 3 oxygen vacancies on the surface. Left: Oxygen-vacancy-defect-free BCF1585. Images generated using CrystalMaker^®^, CrystalMaker Software Ltd., (Oxford, UK), (www.crystalmaker.com, accessed on 26 December 2022).

The results presented here for BCF1585 indicate that once sufficient oxygen vacancies exist on the surface (a surface vacancy concentration of 0.0222 oxygen vacancies per Å^2^), the loss of H_2_ becomes the dominant process. The desorption of H_2_ from the Ba_16_Fe_14_Ce_2_H_2_O_46_ surface of BCF1585 becomes spontaneous (ΔG < 0) above 859 K. Since it was found that from stabilized surfaces of either BCF8515 the structure desorption of H_2_ is spontaneous over all temperatures, H_2_ is expected to more readily desorb from BCF8515 crystallites than from BCF1585. In Figure 5 can be found the oxygen defect free structure and structure with the number of oxygen defects where stabilization was found to begin to occur, for each material investigated. 

### 3.2. Electron Transfers in the Bulk Due to Reductions

Reactions of a metal oxide with diatomic hydrogen are colloquially referred to as reductions, because often the transformation results in a decrease in the formal charge of the metal in the material upon decomposition via dehydration [8,9]. The observed stability of the materials examined in this work implies that the reactions of the surface with hydrogen may not simply be reductions. Bonding in solids can be more complicated than the general definitions of covalent and ionic bonds [28], so the chemical transformations of the materials investigated will herein be discussed in terms of changes in coordination number of the metal sites in the material.

The three possible gas-phase products of reactions (I)–(III) are: H_2(g)_, H_2_O_(g)_, and ½ O_2(g)_, respectively. H_2_ is formed from H atoms on two surface hydroxyl groups. Upon formation of H_2_, the coordination number of each of the associated surface O atoms is reduced by one, provided there is no change in the metal–oxygen coordination for any of the nearest neighbor metal atoms. The loss of ½ O_2_ is equivalent to ejection of a single oxygen atom. The coordination number of each metal atom that the ejected oxygen was coordinated to is reduced by one, provided the metal coordination number does not further change due to surface reconstruction. When a water molecule is lost from the surface, as with the ejection of a single oxygen atom, the metal coordination number of each metal atom that was coordinated to the lost oxygen will decrease in the absence of surface reconstruction.

In the following discussion, the metal coordination changes are tracked for each transformation resulting from reactions (I)–(III) for each of the three structures considered. The coordination changes are used to qualitatively analyze the structural stability of the surface as a whole. This is accomplished by comparing the relative thermodynamic stability of the surface resultant from reactions (I)–(III) with the change in metal coordination on each surface. The thermodynamic ranking for each of reactions (I)–(III) is well represented by the enthalpy change for each respective reaction. Surface reconstruction was observed for some of the resultant surfaces, so the net change in metal coordination was compared to the thermodynamic stability for each surface. The relevant results can be found in Table 1.

#### 3.2.1. Structural Changes of Orthorhombic BCF8515

Analyzing the structural changes resulting from reactions (I)–(III) on the protonated, defect-free surface of the orthorhombic phase of BCF8515 (Ba_16_Ce_14_Fe_2_H_2_O_48_), it was found that reaction (I) results in a net increase in metal coordination of one, reaction (II) results in a net decrease of one metal coordination, and reaction (III) results in a net metal coordination decrease of one due to the loss of an oxygen. Reaction (II) was identified as the transformation with the smallest enthalpy change. Reaction (I) was determined to have the second largest enthalpy change and reaction (III) was found to have the largest enthalpy change. The most negative free energy change was found to be for the reaction that yielded the smallest enthalpy change, as reactions (I)–(III) were endothermic from all surfaces. Comparison of the structural changes caused by each respective reaction to the stability of the resultant structure indicates a decrease in the metal coordination and the loss of a water from the surface yielded the most thermodynamically favorable reaction. The most likely resultant surface starting from a protonated, oxygen-defect-free surface, was found to contain one oxygen vacancy, no surface hydroxyl groups, and a decrease of one metal coordination.

For reactions (I)–(III) from the orthorhombic BCF8515 surface containing one oxygen vacancy (Ba_16_Ce_14_Fe_2_H_2_O_47_), reaction (I) results in no net change of surface metal coordination, reaction (II) results in an increase of one metal coordination, and reaction (III) results in a net decrease of one metal coordination. Reaction (II) was found to have the smallest enthalpy change. Reaction (I) was found to have the second largest enthalpy change. Reaction (III) was found to have the largest enthalpy change. The most thermodynamically favorable resultant surface due to reactions (I)–(III) from orthorhombic BCF8515 with stoichiometry Ba_16_Ce_14_Fe_2_H_2_O_47_ was found to be the surface with stoichiometry Ba_16_Ce_14_Fe_2_O_46_, the surface with two oxygen vacancies and no hydroxyl groups, and an increase in metal coordination of one.

**Figure 6:**
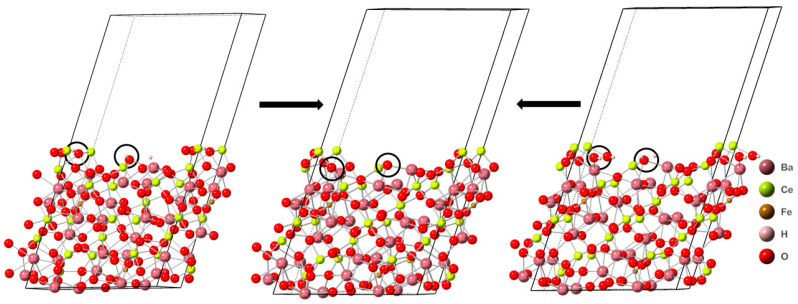
The reaction sites are indicated by black circles. The lefthand structure stoichiometry is Ba_16_Ce_14_Fe_2_H_2_O_47_. The righthand structure stoichiometry is Ba_16_Ce_14_Fe_2_H_2_O_46_. The center structure stoichiometry is Ba_16_Ce_14_Fe_2_O_46_. Images generated using CrystalMaker^®^, CrystalMaker Software Ltd., (Oxford, UK), (www.crystalmaker.com, accessed on 26 December 2022).

Starting from a protonated surface containing two oxygen vacancy defects (Ba_16_Ce_14_Fe_2_H_2_O_46_), reactions (I)–(III) yield a net increase in metal coordination of two for reaction (I), a net decrease in metal coordination of three for reaction (II), and a net decrease of five metal coordinations for reaction (III). Reaction (I) was determined to have the smallest enthalpy change. Reaction (II) was found to have the second largest enthalpy change. Reaction (III) was found to have the largest enthalpy change. Thus, the thermodynamically preferred resultant surface is the result of deprotonation of the two-oxygen-vacancy-containing surface by the formation of H_2_, which results in an increase in the metal coordination. In Figure 6 the transformations that result in the thermodynamically preferred resultant surface can be found.

#### 3.2.2. Structural Changes of Cubic BCF8515

Analyzing structural changes associated with reactions (I)–(III) for the surface of cubic BCF8515 with no surface oxygen vacancies (Ba_16_Ce_14_Fe_2_H_2_O_48_), it was found that reaction (I) results in no net change in metal coordination, reaction (II) results in a net decrease in metal coordination of one, and reaction (III) results in a net increase of one metal coordination. Reaction (II) was found to have the lowest enthalpy change. Reaction (I) had the second lowest enthalpy change and reaction (III) had the highest enthalpy change. The most thermodynamically favorable reaction resulted in the generation of an oxygen vacancy and a net decrease in the metal coordination of the surface.

**Figure 7:**
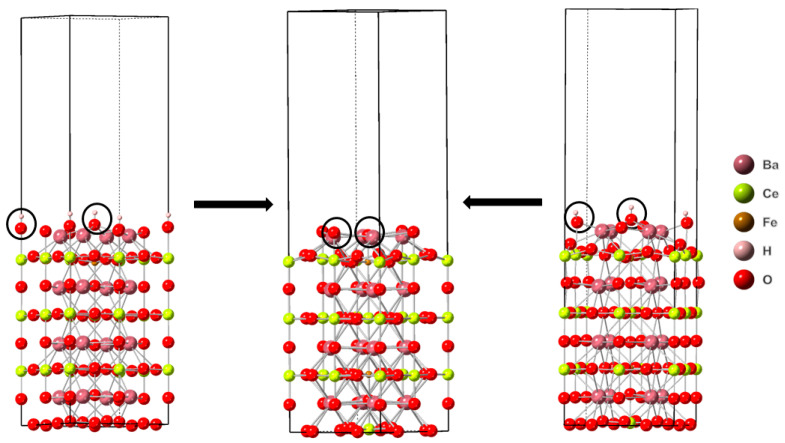
The reaction sites are indicated by black circles. The lefthand structure stoichiometry is Ba_16_Ce_14_Fe_2_H_2_O_48_. The righthand structure stoichiometry is Ba_16_Ce_14_Fe_2_H_2_O_47_. The center structure stoichiometry is Ba_16_Ce_14_Fe_2_O_47_. Images generated using CrystalMaker^®^, CrystalMaker Software Ltd., (Oxford, UK), (www.crystalmaker.com, accessed on 26 December 2022).

Starting from the protonated surface containing one oxygen vacancy (Ba_16_Ce_14_Fe_2_H_2_O_47_), reaction (I) results in a net increase of one metal coordination, reaction (II) results in a net decrease in metal coordination of two, and reaction (III) results in a net decrease in metal coordination of three. Reaction (I) was found to have the smallest enthalpy change. Reaction (II) was found to have the second highest enthalpy change, and reaction (III) was found to result in the largest enthalpy change. The most thermodynamically favorable reaction from the protonated surface with one oxygen vacancy resulted in a net increase in metal coordination and the deprotonation of the surface. In Figure 7 the transformations that result in the thermodynamically preferred resultant surface can be found.

#### 3.2.3. Structural Changes of BCF1585

Analyzing the structural changes that result from reactions (I)–(III) from the protonated defect-free surface of BCF1585 (Ba_16_Fe_14_Ce_2_H_2_O_48_), it was found that reaction (I) results in no net change in the number of metal coordination, reaction (II) results in a net decrease in metal coordination of three, and reaction (III) results in a net decrease in metal coordination of three. Reaction (III) was found to have the smallest enthalpy change. Reaction (II) was found to have the second smallest enthalpy change and reaction (I) was found to have the largest enthalpy change. The thermodynamically most favorable reaction results in the ejection of an oxygen atom, generating an oxygen vacancy on a still-protonated surface, and a net decrease in the metal coordination.

Starting from a protonated surface with one oxygen vacancy (Ba_16_Fe_14_Ce_2_H_2_O_47_), the following structural changes were determined: Reaction (I) results in no changes in the metal coordination. Reaction (II) results in a net decrease in metal coordination of three. Reaction (III) results in a net decrease in metal coordination of seven. The smallest enthalpy change was determined for reaction (II). The second smallest enthalpy change was determined for reaction (III), and the largest enthalpy change was determined for reaction (I). The thermodynamically most favorable reaction generates a deprotonated resultant surface with an additional oxygen vacancy and a net decrease in metal coordination.

**Figure 8:**
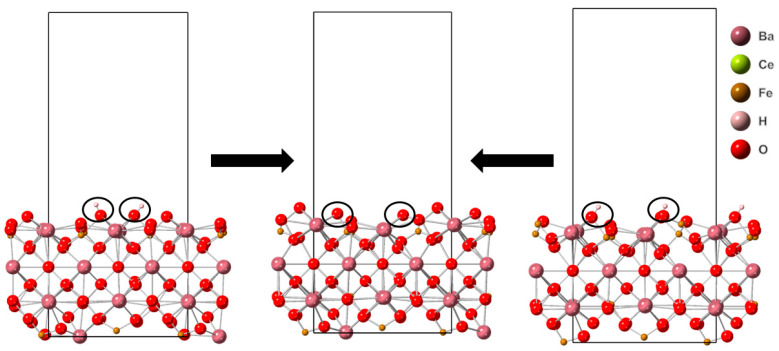
The reaction sites are indicated by white circles. Lefthand structure stoichiometry is Ba_16_Ce_14_Fe_2_H_2_O_47_. The righthand structure stoichiometry is Ba_16_Ce_2_Fe_14_H_2_O_46_. The center structure stoichiometry is Ba_16_Ce_2_Fe_14_O_46_. Images generated using CrystalMaker^®^, CrystalMaker Software Ltd., (Oxford, UK), (www.crystalmaker.com, accessed on 26 December 2022).

Starting from a protonated surface with two oxygen vacancies (Ba_16_Fe_14_Ce_2_H_2_O_46_), the structural changes resulting from reactions (I)–(III) are as follows: Reaction (I) yields a net increase in metal coordination of two. Reaction (II) results in a net decrease in metal coordination of two. Reaction (III) results in a net decrease in metal coordination of one. Reaction (I) was found to have the smallest enthalpy change, while reaction (II) had the second largest, and reaction (III) had the largest enthalpy change. The most thermodynamically favorable reaction results in a structure that is deprotonated, with no additional creation of oxygen vacancies and results in a net increase in metal coordination. In Figure 8 the transformations that result in the thermodynamically preferred resultant surface can be found.

## 4. Discussion

All three structures investigated were found to begin to stabilize once a sufficient concentration of oxygen vacancy defects existed on the surface. Both cubic and orthorhombic BCF8515 structures were determined to lose oxygens from pristine (oxygen-defect-free) surfaces via dehydration. The formation of oxygen vacancies via the loss of ½ O_2_ from the pristine surfaces of BCF8515 was found to not be spontaneous within relevant temperatures. Cubic BCF8515 was found to begin to stabilize at a stoichiometry no richer in oxygen vacancies than Ba_16_Ce_14_Fe_2_H_2_O_47_. Orthorhombic BCF8515 was found to begin to stabilize at a stoichiometry no richer in oxygen vacancies than Ba_16_Ce_14_Fe_2_H_2_O_46_. BCF1585 was found to preferentially form oxygen vacancies via the loss of ½ O_2_ from a pristine surface. From a surface containing one oxygen vacancy per surface cell, the dominant reduction process was found to be the loss of a water. From a surface cell containing two oxygen vacancies, the dominant reduction process for BCF1585 was found to be the loss of H_2_. Thus, BCF1585 was found to begin to stabilize at a stoichiometry no richer in oxygen vacancies than Ba_16_Fe_14_Ce_2_H_2_O_46_.

It is well known that perovskite oxides often form with intrinsic oxygen vacancy defects [6,7,10,13,29,30,31], including BCF8515–BCF1585. For this reason, the stoichiometry for this material is written as BaCe_x_Fe_1-x_O_3-δ_, where x is between 0 and 1, and δ is between 0 and 0.5. From the slab stoichiometries for the stable structures presented above, the minimum δ for each structure was found to be 0.063 for cubic BCF8515, and 0.125 for both orthorhombic BCF8515 and BCF1585. The values for δ calculated here represent an upper limit on the minimum number of oxygen vacancies required for the surface to begin to stabilize. From all three surfaces examined, further loss of water is a spontaneous process at elevated temperatures, but the loss of H_2_ was found to become the dominant process for the δ values presented. The value of δ determined here for BCF1585 is nearly half the value of δ = 0.22 reported by Zhu et al. [13] for the as-synthesized BCF1585, indicating the material formed with a concentration of oxygen vacancies above the minimum required for stabilization to begin.

In all three materials investigated, it was found that while reactions (II) or (III) are the initially dominant processes (reactions that generate oxygen vacancies) the structural changes all result in a decrease in metal–oxygen bonding, and thus a decrease in the metal coordination in the material. Without surface reconstruction, processes that result in the loss of an oxygen reduce the metal coordination in the material. Once the loss of H_2_ becomes the thermodynamically preferred process, an increase in metal coordination (an increase in metal–oxygen bonds) was found to occur in all the materials investigated. These results demonstrate that for a material where the number of oxygen defects is less than the minimum required concentration for stabilization, the metals in the material are electron-poor. Stabilization begins to occur once the metals in the material are in a more favorable (reducing) electronic environment. At the degree of oxygen unsaturation where H_2_ desorbing results in an increase in metal coordination, the enthalpy difference between the protonation and deprotonation becomes small enough that H_2_ desorption becomes dominant. At an oxygen vacancy concentration where protonation decreases metal coordination, deprotonation increases metal coordination, and loss of an oxygen decreases metal coordination, which is more energetically costly than deprotonation. Once this occurs, deprotonation becomes the dominant process, and the material stabilizes.

A common explanation for the intrinsic oxygen-defect nature of this class of materials is the preferred oxidation state of the B-site metals being 3+ [6,13] (oxygen vacancy defects reduce the Ce or Fe to 3+ from 4+). In BaCe_x_Fe_1-x_O_3-δ_, reducing a significant amount of the Fe or Ce to 3+ would result in a significant loss of oxygen from the material, thus the material would not stabilize as rapidly as determined here. This was observed by Zhu et al. [13] in BCF1585. Clearly, the results presented here indicate that the cause of the stability is not entirely due to the complete reduction of all the B-site metals, or a significantly higher oxygen vacancy concentration would be required before the energy required to remove an oxygen becomes greater than that required for deprotonation. These results encourage further investigations into the specific electronic environment that leads to the perovskite oxide stability observed in reducing atmospheres.

From the results obtained for both BCF1585 and BCF8515, water should be observed desorbing from the permeate surface of the binary material as oxygen vacancies form, provided the material as synthesized has a pristine surface with no oxygen defects (an unlikely real-world scenario). Considering the temperatures utilized in the synthesis methods (for calcination and sintering), and the observation that the material contained oxygen vacancy defects prior to testing in the membrane reactor setup [10,12], it is probable that the dehydration of BCF8515-BCF1585 occurs during synthesis, yielding an already-stabilized material prior to use in a membrane reactor system, providing a plausible explanation for why no water was detected by Cheng et al. [10] or Xia et al. [12] in the permeate sweep gas. 

## 5. Conclusions

This theoretical investigation of the stability of the mixed-phase defect perovskite, BCF8515-BCF1585 in the presence of hydrogen reveals that this materials stability is highly dependent on surface structure and the existence of oxygen vacancies on the surface. For each surface investigated, it was found that upon the formation of surface oxygen vacancies, the surfaces begin to stabilize to reduction in a hydrogen-rich environment. The formation of gas-phase species from the protonated surfaces investigated were all entropy-driven processes, while thermodynamic stability was determined largely by the enthalpic changes for each respective reaction. The stability observed arises from the increasing energy required for the removal of successive oxygens. The energy required to reorganize the surface to mitigate the decrease in metal coordination due to the removal of surface oxygens becomes greater than the entropic benefit gained from removing oxygen or water from the surface. Once surface metal coordination decreases enough to make reconstruction too energetically costly, the loss of diatomic hydrogen from the surface becomes the dominant process. This is further supported by the observation that loss of H_2_ from the surface leads to an increase in metal coordination for all the structures investigated. This trend is observed for both the cerium- and iron-dominant phases of the material.

## Figures and Tables

**Table 1:** Enthalpy change and net metal coordination change for each reaction. Bold values indicate the dominant reaction.

		No Oxygen Vacancies Reactant		One Oxygen Vacancy Reactant		Two Oxygen Vacancy Reactant	
Material	Product	Net ∆M-O	∆H (Kcal/mol)	Net ∆M-O	∆H (Kcal/mol)	Net ∆M-O	∆H (Kcal/mol)
Orthorhombic BCF8515	H_2_	+1	82	0	31	**+2**	**13**
	H_2_O	**−1**	**60**	**+1**	**26**	−3	36
	½ O_2_	−1	89	−1	72	−5	128
Cubic BCF8515	H_2_	0	47	**+1**	**6**	N/A	N/A
	H_2_O	**−1**	**34**	−2	37	N/A	N/A
	½ O_2_	+1	88	−3	91	N/A	N/A
BCF1585	H_2_	0	84	0	94	**+2**	**53**
	H_2_O	−3	50	**−2**	**38**	−2	71
	½ O_2_	**−3**	**16**	−7	45	−1	85

## Data Availability

The data presented in this study are available in this article or in the supporting information.

## Supplementary Materials

The following supporting information can be downloaded at: https://www.mdpi.com/article/10.3390/molecules28031429/s1, S1: Bulk Structures and k-points; S2: Orthorhombic BCF8515 slab structures and k-points; S3: Cubic BCF8515 slab structures and k-points, S4: BCF1585 slab structures and k-points, S5: Free energy equations for orthorhombic BCF8515, S6: Free energy equations for cubic BCF8515, S7: Free energy equations for BCF1585.
