# Supplementary material for: The Stability of a Mixed-Phase Barium Cerium Iron Oxide under Reducing Conditions in the Presence of Hydrogen
Source: Molecules. 2023 Feb 2;28(3):1429. doi: 10.3390/molecules28031429 (PMC9920736; doi:10.3390/molecules28031429)
Supplement: Supplementary file 1 [file molecules-28-01429-s001.zip › molecules-2158572-supplementary.pdf]

The Stability of a Mixed-Phase Barium Cerium Iron Oxide Under Reducing Conditions in the Presence of Hydrogen: Supporting Information

Benjamin Rosen and Karl Sohlberg

Note: All lattice vectors are given in Å and all coordinates are fractional.

S1: Bulk Structures

S1.1: Orthorhombic BCF8515 Bulk

K-point grid: 2 1 2

40 ions

0.2234495703708639	0.1970684556695197	-6.1458158332299071
-0.2034790132121650	-12.2742320515120191	-0.3812088266999936
-8.7134635578786970	0.1928833305212641	-0.3010128344501486

Ba	0.49680396	0.47767887	0.26094688
Ba	0.00490182	0.25289991	0.73301291
Ba	0.99093720	0.76207261	0.74766957
Ba	0.50319604	0.02232113	0.73905312
Ba	0.50378433	0.49098395	0.73207619
Ba	-0.00490182	0.24710009	0.26698709
Ba	0.00906280	0.73792739	0.25233043
Ba	0.49621567	0.00901605	0.26792381
Ce	0.50000000	0.25000000	0.00000000
Ce	0.50000000	0.75000000	0.00000000
Ce	-0.00246486	0.00958631	0.50028857
Ce	0.00246486	0.49041369	0.49971143
Ce	-0.00184414	0.00061469	-0.00033538
Ce	0.00184414	0.49938531	0.00033538
Ce	0.50000000	0.75000000	0.50000000
O	0.73798956	0.88650626	0.54331506
O	0.93348309	0.49452305	0.25249159
O	0.28793317	0.60450016	0.04989908
O	0.30071173	0.10270685	0.02444542
O	0.23402839	0.87419294	0.46371046
O	0.27150096	0.35975727	0.48041772
O	0.79855651	0.64743177	0.96915993

O	0.72851864	0.36256392	0.51598615
O	0.57728507	0.76584389	0.25164412
O	0.53712361	0.24755034	0.27546330
O	0.46287639	0.25244966	0.72453670
O	0.06455806	0.48346857	0.74892888
O	0.71206683	0.89549984	0.95010092
O	0.69928827	0.39729315	0.97555458
O	0.76597161	0.62580706	0.53628954
O	0.72849904	0.14024273	0.51958228
O	0.26201044	0.61349374	0.45668494
O	0.06651691	0.00547695	0.74750841
O	0.93544194	0.01653143	0.25107112
O	0.20802011	0.35050886	0.03484639
O	0.20144349	0.85256823	0.03084007
O	0.27148136	0.13743608	0.48401385
O	0.79197989	0.14949114	0.96515361
O	0.42271493	0.73415611	0.74835588
Fe	0.50000000	0.25000000	0.50000000

S1.2: Cubic BCF8515
K-point grid: 2 2 2
40 ions

8.7297543768165795	0.0000000000000000	0.0000000000000000
0.0000000000000000	-8.7297543768165795	0.0000000000000000
0.0000000000000000	0.0000000000000000	-8.7297543768165795

Ba	0.26115529	0.26115529	0.26115529
Ba	0.26115529	0.26115529	0.73884471
Ba	0.26115529	0.73884471	0.26115529
Ba	0.26115529	0.73884471	0.73884471
Ba	0.73884471	0.26115529	0.26115529
Ba	0.73884471	0.26115529	0.73884471
Ba	0.73884471	0.73884471	0.26115529
Ba	0.73884471	0.73884471	0.73884471
Ce	0.00000000	0.00000000	0.00000000
Ce	0.00000000	0.00000000	0.50000000
Ce	0.00000000	0.50000000	0.00000000
Ce	0.00000000	0.50000000	0.50000000
Ce	0.50000000	0.00000000	0.00000000
Ce	0.50000000	0.00000000	0.50000000

Ce	0.50000000	0.50000000	0.00000000
Fe	0.50000000	0.50000000	0.50000000
O	0.00000000	0.25274566	0.00000000
O	0.00000000	0.25366674	0.50000000
O	0.00000000	0.74725434	0.00000000
O	0.00000000	0.74633326	0.50000000
O	0.50000000	0.25366674	0.00000000
O	0.50000000	0.27406529	0.50000000
O	0.50000000	0.74633326	0.00000000
O	0.50000000	0.72593471	0.50000000
O	0.00000000	0.00000000	0.25274566
O	0.00000000	0.00000000	0.74725434
O	0.00000000	0.50000000	0.25366674
O	0.00000000	0.50000000	0.74633326
O	0.50000000	0.00000000	0.25366674
O	0.50000000	0.00000000	0.74633326
O	0.50000000	0.50000000	0.27406529
O	0.50000000	0.50000000	0.72593471
O	0.25274566	0.00000000	0.00000000
O	0.25366674	0.00000000	0.50000000
O	0.25366674	0.50000000	0.00000000
O	0.27406529	0.50000000	0.50000000
O	0.74725434	0.00000000	0.00000000
O	0.74633326	0.00000000	0.50000000
O	0.74633326	0.50000000	0.00000000
O	0.72593471	0.50000000	0.50000000

S1.3: BCF1585

K-point grid: 2 2 2

40 ions

7.9773790556525919	0.0000000000000000	0.0000000000000000
0.0000000000000000	7.9773790556525928	0.0000000000000000
0.0000000000000000	0.0000000000000000	7.9773790556525928

Ba	0.244045258	0.244045258	0.244045258
Ba	0.24404526	0.24404526	0.75595474
Ba	0.24404526	0.75595474	0.24404526
Ba	0.24404526	0.75595474	0.75595474
Ba	0.75595474	0.24404526	0.24404526
Ba	0.75595474	0.24404526	0.75595474
Ba	0.75595474	0.75595474	0.24404526

Ba	0.75595474	0.75595474	0.75595474
Fe	0.00000000	0.00000000	0.00000000
Fe	0.00000000	0.00000000	0.50000000
Fe	0.00000000	0.50000000	0.00000000
Fe	0.00000000	0.50000000	0.50000000
Fe	0.50000000	0.00000000	0.00000000
Fe	0.50000000	0.00000000	0.50000000
Fe	0.50000000	0.50000000	0.00000000
Ce	0.50000000	0.50000000	0.50000000
O	0.00000000	0.24791919	0.00000000
O	0.00000000	0.24731434	0.50000000
O	0.00000000	0.75208081	0.00000000
O	0.00000000	0.75268566	0.50000000
O	0.50000000	0.24731434	0.00000000
O	0.50000000	0.22794956	0.50000000
O	0.50000000	0.75268566	0.00000000
O	0.50000000	0.77205044	0.50000000
O	0.00000000	0.00000000	0.24791919
O	0.00000000	0.00000000	0.75208081
O	0.00000000	0.50000000	0.24731434
O	0.00000000	0.50000000	0.75268566
O	0.50000000	0.00000000	0.24731434
O	0.50000000	0.00000000	0.75268566
O	0.50000000	0.50000000	0.22794956
O	0.50000000	0.50000000	0.77205044
O	0.24791919	0.00000000	0.00000000
O	0.24731434	0.00000000	0.50000000
O	0.24731434	0.50000000	0.00000000
O	0.22794956	0.50000000	0.50000000
O	0.75208081	0.00000000	0.00000000
O	0.75268566	0.00000000	0.50000000
O	0.75268566	0.50000000	0.00000000
O	0.77205044	0.50000000	0.50000000

S2: Orthorhombic BCF8515 slabs

S2.1: No oxygen vacancies, deprotonated

K-point grid: 3 1 1

80 ions

6.1530332564999997   0.0000000000000000   0.0000000000000000

0.0101505200000000	15.0948709692000005	0.0000000000000000
-0.0586197378000000	-9.8991753724000002	28.3526808953999989

Ba	0.51157795	0.11971944	0.31820809
Ba	0.52228637	0.60261523	0.06549189
Ba	0.99509817	0.75994349	0.25378498
Ba	0.94704457	0.27288280	0.01254124
Ba	-0.00641597	0.51087818	0.39644739
Ba	0.02033359	0.00808226	0.11851895
Ba	0.49338860	0.63728008	0.31941883
Ba	0.49669178	0.12185639	0.05289375
Ba	0.50374608	0.40355342	0.44638209
Ba	0.49846134	0.88371022	0.20044469
Ba	0.01165175	-0.01030110	0.37011542
Ba	0.01308604	0.49274486	0.12600419
Ba	0.99093717	0.24279848	0.25269762
Ba	0.04945781	0.79390683	0.00359364
Ba	0.48442444	0.88085274	0.43032718
Ba	0.50636295	0.37235096	0.18992332
Ce	0.51262931	0.12056787	0.43654594
Ce	0.50084878	0.62713708	0.19079654
Ce	0.50229498	0.38058822	0.31562526
Ce	0.53421779	0.87736897	0.06531211
Ce	-0.01217472	0.75381852	0.37647034
Ce	0.00210174	0.24981113	0.12187867
Ce	0.99753517	0.99535114	0.25273085
Ce	0.09352560	0.47994613	-0.01380528
Ce	0.02396587	0.00526497	0.49913026
Ce	0.00184414	0.50047505	0.25018963
Ce	0.00698697	0.25360781	0.38111571
Ce	0.00065358	0.75028629	0.12193055
Ce	0.49402650	0.63755663	0.44770424
Ce	0.49992062	0.12441126	0.18895361
Fe	0.50214659	0.87380176	0.31365378
Fe	0.47786253	0.37054059	0.06853846
O	0.23824195	0.68408832	0.40198556
O	0.27968081	0.15995676	0.13432626
O	0.47290587	-0.01402411	0.36812305
O	0.46500229	0.48907811	0.11939591
O	0.53712362	0.76395649	0.25601891

O	0.52864581	0.27595006	0.01455007
O	0.91837398	0.37006647	0.43419049
O	0.94281269	0.86488028	0.18463798
O	0.30371560	0.46325362	0.27767608
O	0.31493653	0.93934238	0.03090842
O	0.33392337	0.21922991	0.41308871
O	0.28620411	0.69179776	0.14680690
O	0.27397017	0.55149177	0.46312904
O	0.23402840	0.04475876	0.20969331
O	0.27930684	0.81268406	0.33676688
O	0.24692263	0.31556748	0.08551818
O	0.72867976	0.57111687	0.46972438
O	0.73798954	0.07840440	0.23269665
O	0.94365539	0.63392550	0.30983616
O	0.92972194	0.15254887	0.05269850
O	0.00407463	0.89162599	0.43194252
O	0.05999924	0.37978267	0.18030406
O	0.06941417	0.11929074	0.31311080
O	0.20275670	0.61106641	-0.00048414
O	0.78172058	0.14798780	0.39786906
O	0.78460445	0.65790265	0.14564916
O	0.78443447	0.40464940	0.27021007
O	0.79629395	0.92616340	0.03732842
O	0.73008143	0.82975600	0.34091552
O	0.72606228	0.33873980	0.09624621
O	0.21576051	0.07062340	0.45851822
O	0.20802011	0.59216875	0.22156860
O	0.54819035	0.50926261	0.37583944
O	0.58653501	0.98168969	0.13337528
O	0.72839689	0.28275148	0.33476208
O	0.76408498	0.74988944	0.06329707
O	0.71145973	0.04765932	0.47556608
O	0.69928825	0.53913069	0.21843846
O	0.70721671	0.71603941	0.40867306
O	0.77643389	0.18876043	0.16068458
O	0.72358463	0.93779503	0.28514890
O	0.82675167	0.47781887	0.02351171
O	0.21642578	0.34002279	0.34828788
O	0.21723322	0.82240173	0.08762659
O	0.26156596	0.92019602	0.27891894

O	0.27497941	0.43312856	0.03160706
O	0.26727477	0.94275855	0.51718042
O	0.42271492	0.25709990	0.24588299

S2.2: No oxygen vacancies, protonated
K-point grid: 3 1 1
82 ions

6.1530332564999997	0.0000000000000000	0.0000000000000000
0.0101505200000000	15.0948709692000005	0.0000000000000000
-0.0586197378000000	-9.8991753724000002	28.3526808953999989

Ba	0.50856101	0.11619317	0.31749413
Ba	0.53000212	0.59831094	0.06487039
Ba	0.99509817	0.75994349	0.25378498
Ba	0.94494799	0.26830447	0.01260610
Ba	-0.00348334	0.50562342	0.38700208
Ba	0.02073003	0.00971640	0.11196224
Ba	0.49655107	0.63981518	0.31430831
Ba	0.49270117	0.12427551	0.05164896
Ba	0.50796944	0.39602234	0.43932127
Ba	0.49726783	0.88210624	0.19984256
Ba	0.00921263	0.98767373	0.36789138
Ba	0.01226339	0.48989583	0.12456915
Ba	0.99093717	0.24279848	0.25269762
Ba	0.04796443	0.78838771	0.00325543
Ba	0.48937622	0.87327132	0.42268461
Ba	0.50361747	0.37166248	0.18579964
Ce	0.50445918	0.11976925	0.43660730
Ce	0.50050693	0.62470619	0.18803722
Ce	0.49835023	0.37707302	0.31320537
Ce	0.53108734	0.87584605	0.06432685
Ce	0.99511085	0.75491205	0.37570336
Ce	0.00090729	0.25027490	0.12220334
Ce	0.99753517	0.99535114	0.25273085
Ce	0.09717891	0.47671229	0.98739698
Ce	-0.00101656	0.01286967	0.49885219
Ce	0.00184414	0.50047505	0.25018963
Ce	0.00427733	0.25295959	0.38155581
Ce	0.00126514	0.75151344	0.12233519

Ce	0.50688203	0.63384019	0.44340431
Ce	0.50164910	0.12226444	0.18747923
Fe	0.50198057	0.87383377	0.31184714
Fe	0.47758754	0.36777839	0.06692232
O	0.25073257	0.68253504	0.40260349
O	0.28533355	0.16193804	0.13485825
O	0.46601678	0.99256051	0.37255866
O	0.46176674	0.49177695	0.12245419
O	0.53712362	0.76395649	0.25601891
O	0.52838368	0.26602961	0.01444977
O	0.92430143	0.37407099	0.43312314
O	0.94126675	0.86133866	0.18808615
O	0.30408961	0.46635413	0.28029640
O	0.30874845	0.93877593	0.03108164
O	0.33459641	0.21982500	0.41308804
O	0.29510933	0.69642583	0.14644010
O	0.24652168	0.55051683	0.46943338
O	0.23402840	0.04475876	0.20969331
O	0.27054701	0.81097340	0.33786672
O	0.24521124	0.31558630	0.08661938
O	0.74480275	0.57717085	0.46867272
O	0.73798954	0.07840440	0.23269665
O	0.94413419	0.63053363	0.31279451
O	0.93065337	0.14704290	0.05471412
O	0.03977918	0.89497451	0.43737777
O	0.05897341	0.37945776	0.18279449
O	0.06412439	0.12021600	0.31544379
O	0.19940619	0.61052375	0.00290866
O	0.78703763	0.14980567	0.40358451
O	0.79184944	0.65554730	0.14482743
O	0.79411388	0.40885883	0.27656506
O	0.79019945	0.91956003	0.03335535
O	0.73748882	0.82897201	0.34359514
O	0.72659283	0.33584290	0.09676651
O	0.20237751	0.08642750	0.46866156
O	0.20802011	0.59216875	0.22156860
O	0.54015705	0.50479902	0.37945663
O	0.59469603	0.98143649	0.13199233
O	0.71381352	0.28396752	0.33884380
O	0.76108681	0.74919184	0.06356967

O	0.68177477	0.03639166	0.47762153
O	0.69928825	0.53913069	0.21843846
O	0.71667328	0.71702634	0.41047666
O	0.77752112	0.19242561	0.16352086
O	0.72592039	0.93945991	0.28706500
O	0.83459162	0.47044440	0.02636941
O	0.21057830	0.33934849	0.34744267
O	0.20981678	0.82807718	0.08920984
O	0.26377652	0.92211821	0.28010720
O	0.28981846	0.42811973	0.03115467
O	0.13071851	0.96349842	0.55414746
O	0.42271492	0.25709990	0.24588299
H	0.16930126	0.92576125	0.57357881
H	0.10070619	0.55515319	0.48316112

S2.3: 1 oxygen vacancy, deprotonated
K-point grid: 3 1 1
79 ions

6.1530332564999997	0.0000000000000000	0.0000000000000000
0.0101505200000000	15.0948709692000005	0.0000000000000000
-0.0586197378000000	-9.8991753724000002	28.3526808953999989

Ba	0.50642219	0.11763920	0.317199206
Ba	0.52979925	0.59938694	0.065105061
Ba	0.99509817	0.75994349	0.253784984
Ba	0.94510479	0.26948397	0.012662447
Ba	0.99869141	0.50979671	0.396047341
Ba	0.02058613	0.01076903	0.112458695
Ba	0.49812109	0.63427455	0.316130852
Ba	0.49287239	0.12569682	0.052091705
Ba	0.50477359	0.40375456	0.445512196
Ba	0.49715873	0.88240171	0.199179853
Ba	0.00738305	0.98564044	0.365308134
Ba	0.01289012	0.49078388	0.124829874
Ba	0.99093717	0.24279848	0.252697617
Ba	0.04848504	0.78942218	0.003738251
Ba	0.49225720	0.86880658	0.42052978
Ba	0.50571115	0.37175594	0.188092706
Ce	0.50161311	0.12092644	0.437064657

Ce	0.50027189	0.62443785	0.188431983
Ce	0.50111254	0.37921198	0.314955943
Ce	0.53136501	0.87691914	0.064530792
Ce	0.99751193	0.75460515	0.375484311
Ce	0.00113647	0.25137185	0.122334126
Ce	0.99753517	0.99535114	0.252730846
Ce	0.09834375	0.47772595	0.987117869
Ce	-0.00776074	0.02013492	0.498965705
Ce	0.00184414	0.50047505	0.250189632
Ce	0.00213360	0.25306177	0.381805951
Ce	0.00146291	0.75183888	0.1225463
Ce	0.50501642	0.63524508	0.446740152
Ce	0.50127745	0.12355565	0.18783615
Fe	0.50048263	0.87224583	0.310923001
Fe	0.47735788	0.36859760	0.067090256
O	0.25913361	0.69471516	0.403113449
O	0.28520879	0.16283352	0.134907357
O	0.45923108	0.99547940	0.374835672
O	0.46202518	0.49212233	0.121893497
O	0.53712362	0.76395649	0.256018907
O	0.52820527	0.26752005	0.014299488
O	0.92160026	0.36896002	0.434943997
O	0.94022514	0.86195947	0.188043142
O	0.30334012	0.46363543	0.278818571
O	0.30844752	0.93968308	0.031341826
O	0.33592977	0.21688849	0.413307287
O	0.29399476	0.69672406	0.146822079
O	0.27713077	0.55238804	0.462744328
O	0.23402840	0.04475876	0.209693313
O	0.27066117	0.81259753	0.337121966
O	0.24370381	0.31597981	0.085771297
O	0.73569785	0.56818781	0.46941394
O	0.73798954	0.07840440	0.232696652
O	0.94598241	0.63215451	0.31220779
O	0.93052922	0.14839436	0.054675814
O	0.04870677	0.90621965	0.440162093
O	0.06062605	0.38070891	0.1817336
O	0.06324875	0.11805125	0.316147171
O	0.20117181	0.61133921	0.002512163
O	0.79238380	0.14622331	0.406726926

O	0.79110106	0.65653000	0.14515629
O	0.78548219	0.40537493	0.272036456
O	0.79025740	0.92051192	0.033478459
O	0.73545287	0.82996976	0.343632984
O	0.72571251	0.33679986	0.096445393
O	0.19859993	0.10465624	0.481685935
O	0.20802011	0.59216875	0.221568599
O	0.55090157	0.51014524	0.375330742
O	0.59426092	0.98225550	0.132142624
O	0.72175353	0.28116277	0.337101431
O	0.76195119	0.75024877	0.063893578
O	0.67444018	0.03680425	0.482345364
O	0.69928825	0.53913069	0.218438461
O	0.73517148	0.72161617	0.413748052
O	0.77906952	0.19207076	0.163270849
O	0.72432105	0.93954854	0.28704304
O	0.83537840	0.47238721	0.025879209
O	0.21702848	0.33843483	0.349486099
O	0.21107165	0.82849468	0.089593544
O	0.26428096	0.92144418	0.279580935
O	0.29003075	0.42940316	0.030942646
O	0.42271492	0.25709990	0.245882988

S2.4: 1 oxygen vacancy, protonated
K-point grid: 3 1 1
81 ions

6.1530332564999997	0.0000000000000000	0.0000000000000000
0.0101505200000000	15.0948709692000005	0.0000000000000000
-0.0586197378000000	-9.8991753724000002	28.3526808953999989

Ba	0.50115993	0.11482572	0.31512135
Ba	0.53213079	0.59692038	0.06417167
Ba	0.99509817	0.75994349	0.25378498
Ba	0.94533682	0.26792752	0.01234011
Ba	-0.00007883	0.50430601	0.38439592
Ba	0.02003505	0.01028322	0.10904192
Ba	0.49708144	0.63905811	0.31168026
Ba	0.49210924	0.12504755	0.05067718
Ba	0.50761911	0.40060077	0.43923592

Ba	0.49675196	0.88137448	0.19876914
Ba	0.00863972	-0.01458254	0.36462841
Ba	0.01152508	0.48896434	0.12353123
Ba	0.99093717	0.24279848	0.25269762
Ba	0.04623539	0.78611025	0.00297133
Ba	0.49682283	0.87166760	0.42014464
Ba	0.50154086	0.37111413	0.18303902
Ce	0.50825007	0.12735169	0.43874054
Ce	0.50106967	0.62471805	0.18686261
Ce	0.49867429	0.37639107	0.31282929
Ce	0.52914535	0.87536509	0.06362456
Ce	0.00083746	0.75680714	0.37603682
Ce	0.00072967	0.25054755	0.12199344
Ce	0.99753517	0.99535114	0.25273085
Ce	0.09521301	0.47690360	0.98782376
Ce	0.02035081	0.01478187	0.50416378
Ce	0.00184414	0.50047505	0.25018963
Ce	0.00186317	0.25354190	0.38308707
Ce	0.00170378	0.75218267	0.12227233
Ce	0.51745945	0.63585292	0.44318828
Ce	0.50266315	0.12154644	0.18631357
Fe	0.50273004	0.87317374	0.31084176
Fe	0.47831089	0.36731622	0.06612658
O	0.27418631	0.69658142	0.40497988
O	0.28821749	0.16259308	0.13472802
O	0.46740668	0.00332033	0.37793079
O	0.46128341	0.49276476	0.12299244
O	0.53712362	0.76395649	0.25601891
O	0.52918362	0.26370316	0.01402352
O	0.92555728	0.37529818	0.43355524
O	0.94140619	0.86046453	0.18966158
O	0.30329975	0.46731784	0.28177851
O	0.30683474	0.93781482	0.03017620
O	0.34297057	0.22373804	0.41503712
O	0.29985031	0.69903205	0.14646570
O	0.24549040	0.55629648	0.46844280
O	0.23402840	0.04475876	0.20969331
O	0.26705935	0.81313847	0.33832708
O	0.24725603	0.31580529	0.08738267
O	0.74950021	0.57635461	0.46887262

O	0.73798954	0.07840440	0.23269665
O	0.94570266	0.62869414	0.31610236
O	0.93164139	0.14537739	0.05510924
O	0.04914684	0.91277579	0.43986991
O	0.05770230	0.37968377	0.18411676
O	0.05755544	0.12130912	0.31849238
O	0.19423009	0.61235460	0.00573359
O	0.82230082	0.14953150	0.41695468
O	0.79525108	0.65485790	0.14474522
O	0.80073292	0.41040659	0.27996089
O	0.78768232	0.91754986	0.03179771
O	0.74045182	0.82753351	0.34420233
O	0.72771874	0.33454222	0.09693883
O	0.27017211	0.09872735	0.49865314
O	0.20802011	0.59216875	0.22156860
O	0.54346604	0.50796855	0.37878290
O	0.59740758	0.98161352	0.13137386
O	0.70807795	0.28293285	0.34145563
O	0.75826951	0.74981935	0.06383272
O	0.66606615	0.03847968	0.49628430
O	0.69928825	0.53913069	0.21843846
O	0.74137616	0.72911217	0.41913141
O	0.77946449	0.19379747	0.16460265
O	0.72716109	0.94056396	0.28770077
O	0.83514035	0.46773046	0.02753949
O	0.21219718	0.33902612	0.34878197
O	0.20660158	0.83137350	0.08990399
O	0.26621909	0.92422334	0.28050469
O	0.29171937	0.42690950	0.03069731
O	0.42271492	0.25709990	0.24588299
H	0.09735942	0.56055315	0.48117678
H	0.51708753	0.06273157	0.50795361

S2.5: 2 oxygen vacancy, deprotonated
K-point grid: 3 1 1
78 ions

6.1530332564999997	0.0000000000000000	0.0000000000000000
0.0101505200000000	15.0948709692000005	0.0000000000000000
-0.0586197378000000	-9.8991753724000002	28.3526808953999989

Ba	0.49931555	0.11002014	0.31403954
Ba	0.53289141	0.59690556	0.06465605
Ba	0.99509817	0.75994349	0.25378498
Ba	0.94577596	0.26838840	0.01223385
Ba	0.01638992	0.51667537	0.38206760
Ba	0.02032120	0.01097746	0.10837343
Ba	0.50193360	0.63947018	0.31361767
Ba	0.49211586	0.12573291	0.05003776
Ba	0.50299330	0.38549894	0.43630223
Ba	0.49643271	0.88002885	0.19829544
Ba	0.01993615	0.98301792	0.36706881
Ba	0.01268302	0.48937031	0.12364180
Ba	0.99093717	0.24279848	0.25269762
Ba	0.04605802	0.78598230	0.00315018
Ba	0.51437839	0.87456867	0.42174133
Ba	0.50349904	0.37144348	0.18285609
Ce	0.51936344	0.12791562	0.44091848
Ce	0.50100802	0.62508359	0.18754915
Ce	0.50257947	0.38022678	0.31210292
Ce	0.52888853	0.87553482	0.06358984
Ce	0.01148323	0.75549074	0.37758666
Ce	0.00183551	0.25119559	0.12192787
Ce	0.99753517	0.99535114	0.25273085
Ce	0.09530997	0.47795400	0.98817862
Ce	0.03554846	0.01360099	0.50029092
Ce	0.00184414	0.50047505	0.25018963
Ce	0.00214551	0.25165029	0.38121688
Ce	0.00231169	0.75239566	0.12238264
Ce	0.52395941	0.62106401	0.44071552
Ce	0.50378034	0.12162508	0.18577934
Fe	0.51068088	0.87575332	0.31177489
Fe	0.47904361	0.36756268	0.06593750
O	0.27368653	0.68525408	0.40058541
O	0.29173694	0.16314818	0.13440223
O	0.48841992	0.00798624	0.37574600
O	0.46172782	0.49296896	0.12303195
O	0.53712362	0.76395649	0.25601891
O	0.53025443	0.26389200	0.01369224
O	0.75471274	0.22591618	0.42965760

O	0.94253193	0.86074854	0.19021876
O	0.29970898	0.46296433	0.27988819
O	0.30658291	0.93764553	0.02999537
O	0.27787510	0.21161177	0.41881279
O	0.30033104	0.69998241	0.14691196
O	0.28573540	0.53731889	0.45296880
O	0.23402840	0.04475876	0.20969331
O	0.27559105	0.81912928	0.34078277
O	0.24758270	0.31537311	0.08666977
O	0.75132949	0.53007756	0.45296369
O	0.73798954	0.07840440	0.23269665
O	0.95303655	0.62857883	0.31527768
O	0.93135631	0.14536053	0.05533925
O	0.09146828	0.89417363	0.44416544
O	0.06029643	0.38101282	0.18301722
O	0.04313730	0.12198291	0.32163517
O	0.19236536	0.61397819	0.00737514
O	0.80108636	0.04748549	0.45951766
O	0.79569790	0.65583044	0.14527850
O	0.79746700	0.40366963	0.27483344
O	0.78727866	0.91736896	0.03158087
O	0.75183246	0.82929516	0.34480477
O	0.72789900	0.33426973	0.09676772
O	0.32438658	0.07430145	0.49290774
O	0.20802011	0.59216875	0.22156860
O	0.56091049	0.51472549	0.36622507
O	0.59877599	0.98191428	0.13137269
O	0.72297474	0.29350568	0.34589161
O	0.75768423	0.75049386	0.06425065
O	0.69928825	0.53913069	0.21843846
O	0.74855014	0.72268447	0.41855976
O	0.78507334	0.19390238	0.16564789
O	0.73502574	0.94112693	0.28798947
O	0.83512942	0.46734981	0.02758622
O	0.22088654	0.34408734	0.35783142
O	0.20643449	0.83276046	0.09049040
O	0.27223789	0.92857290	0.28147802
O	0.29262909	0.42718323	0.03056785
O	0.42271492	0.25709990	0.24588299

S2.6: 2 oxygen vacancy, protonated

K-point grid: 3 1 1

80 ions

6.1530332564999997	0.0000000000000000	0.0000000000000000
0.0101505200000000	15.0948709692000005	0.0000000000000000
-0.0586197378000000	-9.8991753724000002	28.3526808953999989

Ba	0.49874382	0.10870240	0.31224263
Ba	0.53481391	0.59596035	0.06418996
Ba	0.99509817	0.75994349	0.25378498
Ba	0.94613517	0.26779821	0.01182606
Ba	0.01856567	0.51051379	0.37529017
Ba	0.01976830	0.01076351	0.10719193
Ba	0.49657804	0.64339652	0.31075004
Ba	0.49259479	0.12379510	0.04875226
Ba	0.51724018	0.38405126	0.43432803
Ba	0.49649774	0.87944176	0.19853208
Ba	0.01561060	0.98460959	0.36617122
Ba	0.01305150	0.48843597	0.12288875
Ba	0.99093717	0.24279848	0.25269762
Ba	0.04374447	0.78530565	0.00292848
Ba	0.49995358	0.87725384	0.42544130
Ba	0.50305795	0.37028068	0.17904038
Ce	0.52811978	0.12565512	0.43882646
Ce	0.50275696	0.62553370	0.18659446
Ce	0.50343057	0.37904328	0.31184395
Ce	0.52715470	0.87505486	0.06336724
Ce	0.00706122	0.75477092	0.37728422
Ce	0.00317573	0.25040767	0.12158462
Ce	0.99753517	0.99535114	0.25273085
Ce	0.09189563	0.47919167	0.98912414
Ce	0.02422567	0.02545240	0.50711994
Ce	0.00184414	0.50047505	0.25018963
Ce	0.00922289	0.25119302	0.38209766
Ce	0.00349970	0.75228239	0.12194506
Ce	0.53633120	0.62598077	0.44006005
Ce	0.50447063	0.12073881	0.18492996
Fe	0.50973110	0.87990365	0.31404617
Fe	0.48034600	0.36721465	0.06520470

O	0.28631525	0.68409584	0.40015414
O	0.29478527	0.16292724	0.13375572
O	0.49483215	0.00702530	0.37375342
O	0.46234499	0.49334593	0.12321975
O	0.53712362	0.76395649	0.25601891
O	0.53030744	0.26208485	0.01340493
O	0.76725823	0.22902224	0.43319628
O	0.94406546	0.85908393	0.19113464
O	0.29127426	0.47026501	0.28652512
O	0.30538127	0.93690406	0.02942683
O	0.29557217	0.21634512	0.41894160
O	0.30476067	0.70143071	0.14683241
O	0.25407330	0.53617098	0.46014537
O	0.23402840	0.04475876	0.20969331
O	0.26379050	0.82389266	0.34467080
O	0.25081718	0.31599295	0.08769745
O	0.76514050	0.54422260	0.45787970
O	0.73798954	0.07840440	0.23269665
O	0.94039606	0.62904609	0.31793761
O	0.93228200	0.14435978	0.05533913
O	0.08616772	0.87203109	0.47086897
O	0.06117666	0.37988899	0.18496384
O	0.04365787	0.12262256	0.32221985
O	0.18239673	0.61687853	0.01208941
O	0.80260629	0.03147551	0.45681675
O	0.79922369	0.65457729	0.14506538
O	0.80747280	0.40828305	0.28012087
O	0.78544533	0.91678275	0.03109564
O	0.74914144	0.82940786	0.34854822
O	0.73105384	0.33355401	0.09676576
O	0.30913588	0.05720152	0.48138158
O	0.20802011	0.59216875	0.22156860
O	0.56614794	0.51430570	0.37170562
O	0.60075693	0.98156835	0.13113933
O	0.72496506	0.29769316	0.34870163
O	0.75461534	0.75103782	0.06460162
O	0.69928825	0.53913069	0.21843846
O	0.76257714	0.73898032	0.43025228
O	0.78707992	0.19501023	0.16632882
O	0.73816537	0.94119591	0.28903514

O	0.83302066	0.46382727	0.02816892
O	0.22733306	0.34709079	0.35917354
O	0.20328569	0.83532188	0.09106362
O	0.27324462	0.93031360	0.28245799
O	0.29265878	0.42623187	0.03030215
O	0.42271492	0.25709990	0.24588299
H	0.10543544	0.54909242	0.47275252
H	0.97826057	0.82153053	0.46870422

S2.7: 3 oxygen vacancy, deprotonated
K-point grid: 3 1 1
77 ions

6.1530332564999997	0.0000000000000000	0.0000000000000000
0.0101505200000000	15.0948709692000005	0.0000000000000000
-0.0586197378000000	-9.8991753724000002	28.3526808953999989

Ba	0.49564223	0.11187687	0.31281853
Ba	0.53441143	0.59560792	0.06340167
Ba	0.99509817	0.75994349	0.25378498
Ba	0.94639731	0.26713649	0.01170391
Ba	0.01050175	0.50114057	0.37234381
Ba	0.02058532	0.00986715	0.10736500
Ba	0.48973185	0.64482330	0.30910011
Ba	0.49398111	0.12263390	0.04882425
Ba	0.51731730	0.39612060	0.43973791
Ba	0.49679544	0.88038348	0.19830070
Ba	0.00672143	0.98037792	0.36311503
Ba	0.01283223	0.48804647	0.12229380
Ba	0.99093717	0.24279848	0.25269762
Ba	0.04499422	0.78474917	0.00248864
Ba	0.47837817	0.87565993	0.42145745
Ba	0.50282769	0.36913924	0.17784930
Ce	0.51570471	0.12450031	0.44001932
Ce	0.50295180	0.62482815	0.18578980
Ce	0.50434523	0.37488512	0.31282407
Ce	0.52836456	0.87449764	0.06311457
Ce	0.98465783	0.75763864	0.37736343
Ce	0.00371656	0.24969738	0.12145784
Ce	0.99753517	0.99535114	0.25273085

Ce	0.09314111	0.47730899	0.98817391
Ce	0.02184086	0.04321021	0.49390966
Ce	0.00184414	0.50047505	0.25018963
Ce	0.01444093	0.25877180	0.38339232
Ce	0.00291736	0.75227545	0.12186874
Ce	0.49861489	0.63787253	0.44261629
Ce	0.50450199	0.12065693	0.18515487
Fe	0.50491480	0.87517252	0.31155276
Fe	0.48072018	0.36682550	0.06505963
O	0.24060330	0.68153936	0.40381734
O	0.29315139	0.16220152	0.13375497
O	0.49432110	0.00606902	0.37685494
O	0.46274013	0.49285396	0.12290041
O	0.53712362	0.76395649	0.25601891
O	0.53064229	0.26121634	0.01350005
O	0.94052359	0.38334271	0.43424917
O	0.94376588	0.85974309	0.19045090
O	0.28992790	0.47461124	0.29199321
O	0.30664431	0.93665190	0.02929580
O	0.35415090	0.22231105	0.41518288
O	0.30405421	0.70005604	0.14610902
O	0.23402840	0.04475876	0.20969331
O	0.25735123	0.81540099	0.34092277
O	0.25183558	0.31637482	0.08830307
O	0.41452191	0.56874601	0.48513498
O	0.73798954	0.07840440	0.23269665
O	0.93080885	0.62847027	0.31678251
O	0.93373612	0.14398300	0.05503061
O	0.02136342	0.91463196	0.44064255
O	0.06029662	0.37827538	0.18634539
O	0.04427923	0.12226864	0.32322481
O	0.18751347	0.61421531	0.00899696
O	0.88365767	0.11685839	0.45044873
O	0.79875574	0.65384473	0.14449588
O	0.81118560	0.41318063	0.28513686
O	0.78679148	0.91671647	0.03106300
O	0.73263519	0.82593386	0.34510035
O	0.73289040	0.33361540	0.09667518
O	0.36422034	0.05655676	0.48826811
O	0.20802011	0.59216875	0.22156860

O	0.57163865	0.50371123	0.38607659
O	0.59989291	0.98103926	0.13099226
O	0.70843666	0.28175583	0.34450078
O	0.75637342	0.74993878	0.06384100
O	0.69928825	0.53913069	0.21843846
O	0.72457545	0.73138168	0.42407377
O	0.78321805	0.19484411	0.16529575
O	0.73048358	0.93949556	0.28750641
O	0.83437964	0.46441420	0.02797538
O	0.21287357	0.33550403	0.34767888
O	0.20466480	0.83359344	0.09022529
O	0.26849655	0.92699303	0.28120745
O	0.29268621	0.42534077	0.03006186
O	0.42271492	0.25709990	0.24588299

S2.8: 3 oxygen vacancy, protonated
K-point grid: 3 1 1
79 ions

6.1530332564999997	0.0000000000000000	0.0000000000000000
0.0101505200000000	15.0948709692000005	0.0000000000000000
-0.0586197378000000	-9.8991753724000002	28.3526808953999989

Ba	0.50248925	0.11318835	0.31474879
Ba	0.53435746	0.59523073	0.06296366
Ba	0.99509817	0.75994349	0.25378498
Ba	0.94753140	0.26626775	0.01167935
Ba	0.00992193	0.49406847	0.37188596
Ba	0.02035912	0.00982573	0.10732397
Ba	0.48547380	0.64535994	0.30687811
Ba	0.49486710	0.12242914	0.04912513
Ba	0.52566444	0.38437488	0.43666950
Ba	0.49671913	0.88081345	0.19997980
Ba	0.00211149	0.99084892	0.36663265
Ba	0.01325659	0.48737312	0.12188677
Ba	0.99093717	0.24279848	0.25269762
Ba	0.04453238	0.78474799	0.00251630
Ba	0.46589928	0.87473259	0.42381810
Ba	0.50351190	0.36844318	0.17736736
Ce	0.50597779	0.12057268	0.43727122

Ce	0.50361570	0.62445739	0.18511990
Ce	0.50135403	0.37399348	0.31162333
Ce	0.52808279	0.87431785	0.06334158
Ce	0.98263103	0.75289014	0.37571197
Ce	0.00435037	0.24944155	0.12157099
Ce	0.99753517	0.99535114	0.25273085
Ce	0.09247971	0.47655232	0.98804927
Ce	0.01585261	0.02137238	0.51119654
Ce	0.00184414	0.50047505	0.25018963
Ce	0.00610892	0.25543056	0.38324516
Ce	0.00234834	0.75231581	0.12191152
Ce	0.50245961	0.63697373	0.44116586
Ce	0.50420314	0.11994301	0.18533241
Fe	0.50203382	0.87821453	0.31416140
Fe	0.48160231	0.36648471	0.06489682
O	0.25044427	0.67592430	0.40215564
O	0.29282832	0.16203339	0.13400990
O	0.48407305	0.99760506	0.37492851
O	0.46329120	0.49248021	0.12264690
O	0.53712362	0.76395649	0.25601891
O	0.53152417	0.26064963	0.01342391
O	0.94640184	0.38921488	0.43154829
O	0.94281250	0.85829320	0.19097779
O	0.28844540	0.47679600	0.29235232
O	0.30661049	0.93683437	0.02962182
O	0.32859291	0.21865947	0.41641163
O	0.30442305	0.69982316	0.14589400
O	0.23402840	0.04475876	0.20969331
O	0.24782335	0.81646401	0.34355687
O	0.25322621	0.31594721	0.08837118
O	0.43562401	0.56317451	0.49563076
O	0.73798954	0.07840440	0.23269665
O	0.91977942	0.62831390	0.31725867
O	0.93505996	0.14367054	0.05510830
O	0.03160043	0.87282269	0.46774548
O	0.06091469	0.37819516	0.18650058
O	0.05561287	0.12204821	0.31985350
O	0.18690396	0.61342381	0.00862750
O	0.80389827	0.16129960	0.41473690
O	0.79931050	0.65277684	0.14401315

O	0.81030263	0.41391554	0.28492661
O	0.78601092	0.91651052	0.03112347
O	0.72971360	0.82757410	0.34919447
O	0.73405575	0.33328592	0.09656472
O	0.22729822	0.04574274	0.46644157
O	0.20802011	0.59216875	0.22156860
O	0.56733405	0.50486827	0.38898210
O	0.60062483	0.98048868	0.13111381
O	0.70696381	0.28499643	0.34310942
O	0.75621569	0.74946020	0.06368365
O	0.69928825	0.53913069	0.21843846
O	0.73669781	0.73760872	0.43281015
O	0.78169641	0.19469515	0.16499604
O	0.73174881	0.94074065	0.28946595
O	0.83382189	0.46380019	0.02794778
O	0.21920415	0.33409902	0.34637326
O	0.20327065	0.83327486	0.09007743
O	0.26604286	0.92696262	0.28245505
O	0.29238527	0.42469597	0.02994781
O	0.42271492	0.25709990	0.24588299
H	0.36799438	0.57322751	0.52606057
H	0.92422563	0.82174602	0.46642467

S3: Cubic BCF8515 Slabs

S3.1: No oxygen vacancies, deprotonated

K-point grid: 2 1 2

80 ions

8.7570569223000003	0.0000000000000000	0.0000000000000000
0.0000000000000000	-32.5141138445999971	0.0000000000000000
0.0000000000000000	0.0000000000000000	-8.7570569223000003

Ba	0.25934391	0.06723792	0.25934391
Ba	0.25885388	0.33863710	0.25885388
Ba	0.25934391	0.06723792	0.74065609
Ba	0.25885388	0.33863710	0.74114612
Ba	0.26137730	0.19893395	0.26137730
Ba	0.26343444	0.46224361	0.26343444
Ba	0.26137730	0.19893395	0.73862270
Ba	0.26343444	0.46224361	0.73656556

Ba	0.74065609	0.06723792	0.25934391
Ba	0.74114612	0.33863710	0.25885388
Ba	0.74065609	0.06723792	0.74065609
Ba	0.74114612	0.33863710	0.74114612
Ba	0.73862270	0.19893395	0.26137730
Ba	0.73656556	0.46224361	0.26343444
Ba	0.73862270	0.19893395	0.73862270
Ba	0.73656556	0.46224361	0.73656556
Ce	0.00000000	-0.00062674	0.00000000
Ce	0.00000000	0.26933094	0.00000000
Ce	0.00000000	-0.00141820	0.50000000
Ce	0.00000000	0.26933094	0.50000000
Ce	0.00000000	0.13416315	0.00000000
Ce	0.00000000	0.40808025	0.00000000
Ce	0.00000000	0.13359708	0.50000000
Ce	0.00000000	0.40671263	0.50000000
Ce	0.50000000	-0.00141820	0.00000000
Ce	0.50000000	0.26933094	0.00000000
Ce	0.50000000	0.00410364	0.50000000
Ce	0.50000000	0.26933094	0.50000000
Ce	0.50000000	0.13359708	0.00000000
Ce	0.50000000	0.40671263	0.00000000
Fe	0.50000000	0.13509890	0.50000000
Fe	0.50000000	0.40466112	0.50000000
O	0.00000000	0.06498902	0.00000000
O	0.00000000	0.33857273	0.00000000
O	0.00000000	0.00194570	0.74746845
O	0.00000000	0.26933094	0.74718398
O	0.00000000	0.13439127	0.25315063
O	0.00000000	0.40657832	0.25396546
O	0.00000000	0.13439127	0.74684937
O	0.00000000	0.40657832	0.74603454
O	0.50000000	0.00740133	0.25139967
O	0.50000000	0.26933094	0.25378416
O	0.50000000	0.00740133	0.74860033
O	0.50000000	0.26933094	0.74621584
O	0.50000000	0.13490927	0.27361986
O	0.50000000	0.40460909	0.27326473
O	0.50000000	0.13490927	0.72638014
O	0.50000000	0.40460909	0.72673527

O	0.25253155	0.00194570	0.00000000
O	0.25281602	0.26933094	0.00000000
O	0.25139967	0.00740133	0.50000000
O	0.25378416	0.26933094	0.50000000
O	0.25315063	0.13439127	0.00000000
O	0.25396546	0.40657832	0.00000000
O	0.00000000	0.06561690	0.50000000
O	0.00000000	0.33858604	0.50000000
O	0.27361986	0.13490927	0.50000000
O	0.27326473	0.40460909	0.50000000
O	0.74746845	0.00194570	0.00000000
O	0.74718398	0.26933094	0.00000000
O	0.74860033	0.00740133	0.50000000
O	0.74621584	0.26933094	0.50000000
O	0.74684937	0.13439127	0.00000000
O	0.74603454	0.40657832	0.00000000
O	0.72638014	0.13490927	0.50000000
O	0.72673527	0.40460909	0.50000000
O	0.00000000	0.20123977	0.00000000
O	0.00000000	0.47290318	0.00000000
O	0.00000000	0.20097902	0.50000000
O	0.00000000	0.47273850	0.50000000
O	0.50000000	0.06561690	0.00000000
O	0.50000000	0.33858604	0.00000000
O	0.50000000	0.07306573	0.50000000
O	0.50000000	0.34318118	0.50000000
O	0.50000000	0.20097902	0.00000000
O	0.50000000	0.47273850	0.00000000
O	0.50000000	0.19546987	0.50000000
O	0.50000000	0.46272029	0.50000000
O	0.00000000	0.00194570	0.25253155
O	0.00000000	0.26933094	0.25281602

S3.2: No oxygen vacancies, protonated

K-point grid: 2 1 2

82 ions

8.7570569223000003	0.0000000000000000	0.0000000000000000
0.0000000000000000	-32.5141138445999971	0.0000000000000000
0.0000000000000000	0.0000000000000000	-8.7570569223000003

Ba	0.26117123	0.06786246	0.26116931
Ba	0.26324238	0.33718206	0.26254707
Ba	0.26121539	0.06786203	0.73882865
Ba	0.26237180	0.33700015	0.73779339
Ba	0.26137730	0.19893395	0.26137730
Ba	0.26749939	0.46254403	0.27031809
Ba	0.26137730	0.19893395	0.73862270
Ba	0.26671439	0.46281675	0.73465533
Ba	0.73882718	0.06786259	0.26118420
Ba	0.73937386	0.33668822	0.26088189
Ba	0.73882008	0.06786192	0.73877228
Ba	0.74004022	0.33650905	0.73969107
Ba	0.73862270	0.19893395	0.26137730
Ba	0.73372311	0.46271674	0.26799511
Ba	0.73862270	0.19893395	0.73862270
Ba	0.73421516	0.46303650	0.73731209
Ce	0.00004291	0.99930001	0.00000541
Ce	0.00000000	0.26933094	0.00000000
Ce	1.00001148	0.99865840	0.49998779
Ce	0.00000000	0.26933094	0.50000000
Ce	0.00003391	0.13356781	0.00000462
Ce	0.99957080	0.40683707	0.00083534
Ce	0.00001586	0.13325619	0.49998327
Ce	0.00043428	0.40768981	0.50111262
Ce	0.50002305	0.99865982	0.99997975
Ce	0.50000000	0.26933094	0.00000000
Ce	0.50001093	0.00442136	0.49997976
Ce	0.50000000	0.26933094	0.50000000
Ce	0.50000413	0.13325595	0.99999254
Ce	0.49991036	0.40769789	0.00072091
Fe	0.50002709	0.13491330	0.50000251
Fe	0.50138385	0.39864446	0.50149111
O	0.49919969	0.52092736	0.50039715
O	0.00611041	0.51329985	0.00390704
O	0.00038123	0.06495189	0.00026087
O	0.99316385	0.34007896	0.99778917
O	0.00003462	0.00146794	0.74739068
O	0.00000000	0.26933094	0.74718398
O	0.00021991	0.13556360	0.25232645

O	0.99866861	0.41115432	0.24904049
O	0.99985626	0.13547758	0.74766356
O	0.00017433	0.41256520	0.75265054
O	0.50005294	0.00806492	0.25134691
O	0.50000000	0.26933094	0.25378416
O	0.50002948	0.00805054	0.74860779
O	0.50000000	0.26933094	0.74621584
O	0.49995490	0.13521010	0.27000275
O	0.50145239	0.40637228	0.27394121
O	0.50012605	0.13521440	0.72998659
O	0.50067928	0.40590967	0.72805829
O	0.25264461	0.00134363	0.99998360
O	0.25281602	0.26933094	0.00000000
O	0.25138385	0.00806556	0.49997973
O	0.25378416	0.26933094	0.50000000
O	0.25235217	0.13556461	0.99978737
O	0.24787064	0.41015624	0.00281627
O	0.99996563	0.06556838	0.49976455
O	0.00035215	0.34005096	0.50240890
O	0.27003890	0.13520474	0.50009044
O	0.27386009	0.40558578	0.50117388
O	0.74743939	0.00147650	0.99996459
O	0.74718398	0.26933094	0.00000000
O	0.74864457	0.00804894	0.50000810
O	0.74621584	0.26933094	0.50000000
O	0.74769105	0.13546499	0.00015490
O	0.75151057	0.41347334	0.00139978
O	0.73001951	0.13521819	0.49991775
O	0.72797244	0.40670570	0.50193908
O	0.00000000	0.20123977	0.00000000
O	0.99704970	0.48361910	0.99523031
O	0.00000000	0.20097902	0.50000000
O	0.00814181	0.47357553	0.49685911
O	0.49978541	0.06556825	0.99993696
O	0.50347918	0.34004752	0.00178306
O	0.50006201	0.07326243	0.50001204
O	0.50070224	0.34121175	0.49972726
O	0.50000000	0.20097902	0.00000000
O	0.49473933	0.47358877	0.99709537
O	0.50000000	0.19546987	0.50000000

O	0.49963059	0.49092244	0.49869099
H	1.00000669	0.00135259	0.25260044
H	0.00000000	0.26933094	0.25281602

S3.3: 1 oxygen vacancy, deprotonated
K-point grid: 2 1 2
79 ions

8.7570569223000003	0.0000000000000000	0.0000000000000000
0.0000000000000000	-32.5141138445999971	0.0000000000000000
0.0000000000000000	0.0000000000000000	-8.7570569223000003

Ba	0.26097094	0.06812936	0.26097094
Ba	0.26898059	0.33676001	0.26898059
Ba	0.26097094	0.06812936	0.73902906
Ba	0.26898059	0.33676001	0.73101941
Ba	0.26137730	0.19893395	0.26137730
Ba	0.28013145	0.46220180	0.28013145
Ba	0.26137730	0.19893395	0.73862270
Ba	0.28013145	0.46220180	0.71986855
Ba	0.73902906	0.06812936	0.26097094
Ba	0.73101941	0.33676001	0.26898059
Ba	0.73902906	0.06812936	0.73902906
Ba	0.73101941	0.33676001	0.73101941
Ba	0.73862270	0.19893395	0.26137730
Ba	0.71986855	0.46220180	0.28013145
Ba	0.73862270	0.19893395	0.73862270
Ba	0.71986855	0.46220180	0.71986855
Ce	0.00000000	0.99934337	0.00000000
Ce	0.00000000	0.26933094	0.00000000
Ce	0.00000000	0.99883922	0.50000000
Ce	0.00000000	0.26933094	0.50000000
Ce	0.00000000	0.13367755	0.00000000
Ce	0.00000000	0.40456042	0.00000000
Ce	0.00000000	0.13347666	0.50000000
Ce	0.00000000	0.40896519	0.50000000
Ce	0.50000000	0.99883922	0.00000000
Ce	0.50000000	0.26933094	0.00000000
Ce	0.50000000	0.00498121	0.50000000
Ce	0.50000000	0.26933094	0.50000000

Ce	0.50000000	0.13347666	0.00000000
Ce	0.50000000	0.40896519	0.00000000
Fe	0.50000000	0.13561495	0.50000000
Fe	0.50000000	0.40323198	0.50000000
O	0.00000000	0.06496248	0.00000000
O	0.00000000	0.33979591	0.00000000
O	0.00000000	0.00145805	0.74732490
O	0.00000000	0.26933094	0.74718398
O	0.00000000	0.13529212	0.25237344
O	0.00000000	0.41927997	0.24355084
O	0.00000000	0.13529212	0.74762656
O	0.00000000	0.41927997	0.75644916
O	0.50000000	0.00840464	0.25142487
O	0.50000000	0.26933094	0.25378416
O	0.50000000	0.00840464	0.74857513
O	0.50000000	0.26933094	0.74621584
O	0.50000000	0.13596903	0.27054061
O	0.50000000	0.40387583	0.26741739
O	0.50000000	0.13596903	0.72945939
O	0.50000000	0.40387583	0.73258261
O	0.25267510	0.00145805	0.00000000
O	0.25281602	0.26933094	0.00000000
O	0.25142487	0.00840464	0.50000000
O	0.25378416	0.26933094	0.50000000
O	0.25237344	0.13529212	0.00000000
O	0.24355084	0.41927997	0.00000000
O	0.00000000	0.06576516	0.50000000
O	0.00000000	0.34075267	0.50000000
O	0.27054061	0.13596903	0.50000000
O	0.26741739	0.40387583	0.50000000
O	0.74732490	0.00145805	0.00000000
O	0.74718398	0.26933094	0.00000000
O	0.74857513	0.00840464	0.50000000
O	0.74621584	0.26933094	0.50000000
O	0.74762656	0.13529212	0.00000000
O	0.75644916	0.41927997	0.00000000
O	0.72945939	0.13596903	0.50000000
O	0.73258261	0.40387583	0.50000000
O	0.00000000	0.20123977	0.00000000
O	0.00000000	0.20097902	0.50000000

O	0.00000000	0.47463043	0.50000000
O	0.50000000	0.06576516	0.00000000
O	0.50000000	0.34075267	0.00000000
O	0.50000000	0.07397184	0.50000000
O	0.50000000	0.34139021	0.50000000
O	0.50000000	0.20097902	0.00000000
O	0.50000000	0.47463043	0.00000000
O	0.50000000	0.19800601	0.50000000
O	0.50000000	0.46626182	0.50000000
O	0.00000000	0.00145805	0.25267510
O	0.00000000	0.26933094	0.25281602

S3.4: 1 oxygen vacancy, protonated

K-point grid: 2 1 2

81 ions

8.7570569223000003	0.0000000000000000	0.0000000000000000
0.0000000000000000	-32.5141138445999971	0.0000000000000000
0.0000000000000000	0.0000000000000000	-8.7570569223000003

Ba	0.26129695	0.06811192	0.26122195
Ba	0.26015838	0.33402531	0.26399023
Ba	0.26138477	0.06810511	0.73861660
Ba	0.27688267	0.33863793	0.72914215
Ba	0.26137730	0.19893395	0.26137730
Ba	0.28540916	0.46511751	0.26173671
Ba	0.26137730	0.19893395	0.73862270
Ba	0.26964600	0.46256501	0.72561247
Ba	0.73871927	0.06811449	0.26120317
Ba	0.74092480	0.33296436	0.26030248
Ba	0.73863767	0.06810365	0.73864048
Ba	0.72392386	0.33764651	0.73090683
Ba	0.73862270	0.19893395	0.26137730
Ba	0.71261466	0.46566828	0.26040392
Ba	0.73862270	0.19893395	0.73862270
Ba	0.72974778	0.46330191	0.72294782
Ce	-0.00000472	0.99918636	-0.00001534
Ce	0.00000000	0.26933094	0.00000000
Ce	0.00001072	0.99874023	0.49993075
Ce	0.00000000	0.26933094	0.50000000

Ce	-0.00000426	0.13338504	-0.00000531
Ce	-0.00214273	0.40598169	0.00509659
Ce	0.00000239	0.13333230	0.49993122
Ce	-0.00115839	0.40801925	0.48976052
Ce	0.50000588	0.99873468	-0.00009358
Ce	0.50000000	0.26933094	0.00000000
Ce	0.50001169	0.00498800	0.49990313
Ce	0.50000000	0.26933094	0.50000000
Ce	0.50000942	0.13333413	-0.00006150
Ce	0.50084318	0.40850152	0.00102493
Fe	0.50001176	0.13565380	0.49989170
Fe	0.50036708	0.40008200	0.49576968
O	0.49729597	0.52278576	0.50165721
O	0.00059664	0.50942414	0.48829846
O	-0.00013503	0.06484126	0.00063967
O	-0.01018757	0.34140647	0.04633586
O	0.00000632	0.00149591	0.74732537
O	0.00000000	0.26933094	0.74718398
O	-0.00001946	0.13573277	0.25201567
O	-0.00074632	0.43429348	0.24548642
O	-0.00000943	0.13550902	0.74791174
O	-0.00130965	0.41450927	0.74689537
O	0.49999855	0.00845170	0.25118451
O	0.50000000	0.26933094	0.25378416
O	0.50000221	0.00839206	0.74860952
O	0.50000000	0.26933094	0.74621584
O	0.50001925	0.13621558	0.26941295
O	0.50025348	0.40375837	0.26858889
O	0.50001319	0.13628151	0.73041904
O	0.50077755	0.40790293	0.72912606
O	0.25260474	0.00139555	-0.00009197
O	0.25281602	0.26933094	0.00000000
O	0.25127299	0.00842330	0.49994714
O	0.25378416	0.26933094	0.50000000
O	0.25204463	0.13561351	-0.00000269
O	0.24433434	0.41713082	-0.00864731
O	0.00005143	0.06567561	0.49939613
O	0.00183488	0.34145687	0.47094667
O	0.26949161	0.13626068	0.49986831
O	0.26641787	0.40740478	0.49454285

O	0.74739427	0.00134510	-0.00009363
O	0.74718398	0.26933094	0.00000000
O	0.74875188	0.00843214	0.49994434
O	0.74621584	0.26933094	0.50000000
O	0.74795955	0.13565658	0.00000429
O	0.75547535	0.42287417	-0.00787796
O	0.73052155	0.13624907	0.49986622
O	0.73255832	0.40781444	0.49340269
O	0.00000000	0.20123976	0.00000000
O	0.00000000	0.20097902	0.50000000
O	0.00018583	0.48579308	0.55690905
O	0.50011084	0.06567980	-0.00030387
O	0.50725010	0.34071199	-0.01869259
O	0.49998784	0.07402215	0.50003856
O	0.49894240	0.34126430	0.50739618
O	0.50000000	0.20097902	0.00000000
O	0.49241131	0.47496887	0.04272925
O	0.50003749	0.19890435	0.49967580
O	0.49730926	0.49273639	0.50525018
H	0.00001063	0.00122264	0.25260102
H	0.00000000	0.26933094	0.25281602

S3.5: 2 oxygen vacancy, deprotonated
K-point grid: 2 1 2
78 ions

8.7570569223000003	0.0000000000000000	0.0000000000000000
0.0000000000000000	-32.5141138445999971	0.0000000000000000
0.0000000000000000	0.0000000000000000	-8.7570569223000003

Ba	0.26110073	0.06805989	0.26112976
Ba	0.26694041	0.33586893	0.27230364
Ba	0.26110073	0.06805989	0.73887024
Ba	0.26694041	0.33586893	0.72769636
Ba	0.26137730	0.19893395	0.26137730
Ba	0.27109458	0.46320797	0.29161503
Ba	0.26137730	0.19893395	0.73862270
Ba	0.27109458	0.46320797	0.70838497
Ba	0.73889927	0.06805989	0.26112976
Ba	0.73305959	0.33586893	0.27230364
Ba	0.73889927	0.06805989	0.73887024

Ba	0.73305959	0.33586893	0.72769636
Ba	0.73862270	0.19893395	0.26137730
Ba	0.72890542	0.46320797	0.29161503
Ba	0.73862270	0.19893395	0.73862270
Ba	0.72890542	0.46320797	0.70838497
Ce	0.00000000	0.99922296	0.00000000
Ce	0.00000000	0.26933094	0.00000000
Ce	0.00000000	0.99874026	0.50000000
Ce	0.00000000	0.26933094	0.50000000
Ce	0.00000000	0.13345462	0.00000000
Ce	0.00000000	0.40798627	0.00000000
Ce	0.00000000	0.13333993	0.50000000
Ce	0.00000000	0.40681584	0.50000000
Ce	0.50000000	0.99872601	0.00000000
Ce	0.50000000	0.26933094	0.00000000
Ce	0.50000000	0.00496108	0.50000000
Ce	0.50000000	0.26933094	0.50000000
Ce	0.50000000	0.13330824	0.00000000
Ce	0.50000000	0.40729606	0.00000000
Fe	0.50000000	0.13563085	0.50000000
Fe	0.50000000	0.40401368	0.50000000
O	0.00000000	0.06488720	0.00000000
O	0.00000000	0.34252321	0.00000000
O	0.00000000	0.00143628	0.74735780
O	0.00000000	0.26933094	0.74718398
O	0.00000000	0.13561590	0.25210504
O	0.00000000	0.41592089	0.24474513
O	0.00000000	0.13561590	0.74789496
O	0.00000000	0.41592089	0.75525487
O	0.50000000	0.00841008	0.25134056
O	0.50000000	0.26933094	0.25378416
O	0.50000000	0.00841008	0.74865944
O	0.50000000	0.26933094	0.74621584
O	0.50000000	0.13618859	0.26973239
O	0.50000000	0.40633510	0.26151949
O	0.50000000	0.13618859	0.73026761
O	0.50000000	0.40633510	0.73848051
O	0.25264163	0.00143216	0.00000000
O	0.25281602	0.26933094	0.00000000
O	0.25134118	0.00841970	0.50000000

O	0.25378416	0.26933094	0.50000000
O	0.25213024	0.13563158	0.00000000
O	0.25415933	0.43251084	0.00000000
O	0.00000000	0.06567350	0.50000000
O	0.00000000	0.33936874	0.50000000
O	0.26977505	0.13619379	0.50000000
O	0.26614184	0.40339276	0.50000000
O	0.74735837	0.00143216	0.00000000
O	0.74718398	0.26933094	0.00000000
O	0.74865882	0.00841970	0.50000000
O	0.74621584	0.26933094	0.50000000
O	0.74786976	0.13563158	0.00000000
O	0.74584067	0.43251084	0.00000000
O	0.73022495	0.13619379	0.50000000
O	0.73385816	0.40339276	0.50000000
O	0.00000000	0.20123977	0.00000000
O	0.00000000	0.20097902	0.50000000
O	0.00000000	0.47377947	0.50000000
O	0.50000000	0.06566351	0.00000000
O	0.50000000	0.34152627	0.00000000
O	0.50000000	0.07399727	0.50000000
O	0.50000000	0.34139011	0.50000000
O	0.50000000	0.20097902	0.00000000
O	0.50000000	0.19870356	0.50000000
O	0.50000000	0.46778848	0.50000000
O	0.00000000	0.00143628	0.25264220
O	0.00000000	0.26933094	0.25281602

S3.6: 2 oxygen vacancies, protonated

K-point grid: 2 1 2

80 ions

8.7570569223000003	0.0000000000000000	0.0000000000000000
0.0000000000000000	-32.5141138445999971	0.0000000000000000
0.0000000000000000	0.0000000000000000	-8.7570569223000003

Ba	0.26292905	0.06464617	0.26462871
Ba	0.25596330	0.32959076	0.25514771
Ba	0.26343808	0.06618207	0.74324081
Ba	0.27436182	0.33630820	0.72468822

Ba	0.26352872	0.19133053	0.27850383
Ba	0.27477178	0.46509275	0.27598900
Ba	0.26137730	0.19893395	0.73862270
Ba	0.26793300	0.46416524	0.71702368
Ba	0.73889927	0.06805989	0.26112976
Ba	0.74366945	0.32972056	0.25535153
Ba	0.74029969	0.06812830	0.73959533
Ba	0.72560336	0.33614919	0.72516343
Ba	0.72643206	0.19245278	0.27289030
Ba	0.72533901	0.46511628	0.27595122
Ba	0.73862270	0.19893395	0.73862270
Ba	0.73204652	0.46413771	0.71697447
Ce	0.00000000	0.99922296	0.00000000
Ce	-0.00007783	0.26687113	0.00067233
Ce	0.00217467	0.99831768	0.50544432
Ce	0.00000000	0.26933094	0.50000000
Ce	-0.00131988	0.13249411	0.00060733
Ce	0.00000000	0.40798627	0.00000000
Ce	0.00033884	0.13205217	0.50779612
Ce	0.00001315	0.40661259	0.49058800
Ce	0.50278610	0.99777424	0.00380948
Ce	0.49969376	0.26696737	0.00015106
Ce	0.50322635	0.00359832	0.50417703
Ce	0.50000000	0.26933094	0.50000000
Ce	0.50043335	0.13216078	-0.00109521
Ce	0.50000000	0.40729606	0.00000000
Fe	0.49976064	0.13346274	0.50617534
Fe	0.50006709	0.40094049	0.49579361
O	0.00003155	0.51305868	0.49034429
O	0.50004509	0.52395239	0.50293652
O	-0.01688416	0.06466694	0.96292974
O	-0.00010129	0.34305157	0.03773121
O	0.01060050	-0.00995945	0.75083041
O	0.00000000	0.26933094	0.74718398
O	0.03456899	0.12865827	0.25543904
O	0.00004356	0.43159858	0.24595897
O	0.95625450	0.13990823	0.75242584
O	0.00009033	0.41606791	0.74717008
O	0.50671861	0.00579669	0.25478650
O	0.50000000	0.26933094	0.25378416

O	0.49729945	0.00711549	0.75373732
O	0.50000000	0.26933094	0.74621584
O	0.48112464	0.13449359	0.26937412
O	0.50012461	0.40966545	0.26370833
O	0.52127236	0.13432687	0.73521985
O	0.50002062	0.40807883	0.73292217
O	0.25348560	0.00646647	0.01036251
O	0.25281602	0.26933094	0.00000000
O	0.25323160	0.00569804	0.49685929
O	0.25378416	0.26933094	0.50000000
O	0.25055523	0.13289835	-0.03548196
O	0.25206719	0.43054887	0.99684130
O	0.00913841	0.06499119	0.54256262
O	0.00037279	0.34047280	0.47864855
O	0.26705539	0.13578565	0.52530477
O	0.26633721	0.40747303	0.49515866
O	0.74982407	-0.00404032	0.00165914
O	0.74718398	0.26933094	0.00000000
O	0.75256724	0.00854709	0.50804044
O	0.74621584	0.26933094	0.50000000
O	0.74868357	0.13566551	0.04460438
O	0.74796033	0.43057974	0.99685757
O	0.73326687	0.13283939	0.48462013
O	0.73372759	0.40743288	0.49506362
O	0.00000000	0.20123977	0.00000000
O	0.00000000	0.20097902	0.50000000
O	-0.00002123	0.48641968	0.54147504
O	0.51853252	0.06499862	0.00709698
O	0.50015291	0.34131932	0.99234638
O	0.49670454	0.07229688	0.50292204
O	0.50001935	0.34085860	0.50478801
O	0.50000000	0.20097902	0.00000000
O	0.50072086	0.19762354	0.50376321
O	0.49999922	0.49390598	0.50362940
H	-0.00157969	0.01008134	0.25521255
H	0.00000000	0.26933094	0.25281602

S4: BCF1585 Slabs

S4.1: No oxygen vacancies, deprotonated

K-point grid: 2 2 1

80 ions

11.2817173004000004	0.0000000000000000	0.0000000000000000
0.0000000000000000	7.9773788451999996	0.0000000000000000
0.0000000000000000	0.0000000000000000	26.2817173004000004

Ba	0.00542702	0.75213573	0.10805084
Ba	0.49634216	0.76873579	0.32324218
Ba	0.25595474	0.75595474	0.21463051
Ba	0.75596636	0.74294451	0.99347723
Ba	0.00542702	0.24786427	0.10805084
Ba	0.49634216	0.23126421	0.32324218
Ba	0.25595474	0.24404526	0.21463051
Ba	0.75596636	0.25705549	0.99347723
Ba	0.74404520	0.75595474	0.21463051
Ba	0.24364208	0.73953083	0.99789236
Ba	0.99532728	0.76054419	0.33318104
Ba	0.50546954	0.73531941	0.11048958
Ba	0.74404520	0.24404526	0.21463051
Ba	0.24364208	0.26046917	0.99789236
Ba	0.99532728	0.23945581	0.33318104
Ba	0.50546954	0.26468059	0.11048958
Fe	0.99948329	0.00000000	0.01518874
Fe	0.50000000	0.00000000	0.21463051
Fe	0.26035869	0.00000000	0.11203231
Fe	0.76414712	0.00000000	0.33196798
Fe	0.99355179	0.50000000	0.99595689
Fe	0.50000000	0.50000000	0.21463051
Fe	0.25443847	0.50000000	0.10441728
Fe	0.73415330	0.50000000	0.31406915
Fe	0.75074632	0.00000000	0.10885204
Fe	0.22520440	0.00000000	0.33592171
Fe	0.00000000	0.00000000	0.21463051
Fe	0.50109412	0.00000000	0.01748246
Fe	0.76520431	0.50000000	0.09814190
Fe	0.25744044	0.50000000	0.31668964
Ce	0.00000000	0.50000000	0.21463051
Ce	0.49949307	0.50000000	0.97798274
O	0.99888029	0.80721051	0.98635729
O	0.50000000	0.75208080	0.21463051

O	0.25424373	0.75242335	0.10654329
O	0.74108060	0.69210816	0.34241887
O	0.99888029	0.19278949	0.98635729
O	0.50000000	0.24791920	0.21463051
O	0.25424373	0.24757665	0.10654329
O	0.74108060	0.30789184	0.34241887
O	0.75393794	0.74538554	0.10402574
O	0.25280037	0.69046549	0.34560816
O	0.00000000	0.77205044	0.21463051
O	0.50056454	0.80799695	0.98744003
O	0.75393794	0.25461446	0.10402574
O	0.25280037	0.30953451	0.34560816
O	0.00000000	0.22794956	0.21463051
O	0.50056454	0.19200305	0.98744003
O	0.13258446	0.00000000	0.04985002
O	0.61921624	0.00000000	0.26201877
O	0.37604040	0.00000000	0.16141950
O	0.87108934	0.00000000	0.37663201
O	0.13202667	0.50000000	0.05598224
O	0.60723059	0.50000000	0.27535414
O	0.37634283	0.50000000	0.16154932
O	0.88304749	0.50000000	0.95331828
O	0.87365711	0.00000000	0.16039647
O	0.35648822	0.00000000	0.36416412
O	0.13494597	0.00000000	0.27825148
O	0.63828907	0.00000000	0.04960883
O	0.86397487	0.50000000	0.15624020
O	0.34745943	0.50000000	0.94084923
O	0.12676440	0.50000000	0.28079756
O	0.64257610	0.50000000	0.05043757
O	0.86726301	0.00000000	0.05024676
O	0.38554641	0.00000000	0.26339903
O	0.12634283	0.00000000	0.16039647
O	0.63476632	0.00000000	0.36245174
O	0.88838945	0.50000000	0.05228813
O	0.37866100	0.50000000	0.27519479
O	0.13602522	0.50000000	0.15624020
O	0.64973814	0.50000000	0.93911203
O	0.62828103	0.00000000	0.15563999
O	0.11691059	0.00000000	0.37958088

O	0.86597839	0.00000000	0.27605008
O	0.36807394	0.00000000	0.05276310
O	0.62080944	0.50000000	0.16910213
O	0.11156969	0.50000000	0.95832150
O	0.86604184	0.50000000	0.27838627
O	0.37218369	0.50000000	0.05319636

S4.2: No oxygen vacancies, protonated

K-point grid: 2 2 1

82 ions

11.2817173004000004	0.0000000000000000	0.0000000000000000
0.0000000000000000	7.9773788451999996	0.0000000000000000
0.0000000000000000	0.0000000000000000	26.2817173004000004

H	0.31968052	0.01136879	0.40134054
H	0.27957688	0.74702333	0.36630873
Ba	0.00405351	0.75150707	0.10784949
Ba	0.50351452	0.77130236	0.32101619
Ba	0.25595474	0.75595474	0.21463051
Ba	0.72495311	0.75481809	0.99448438
Ba	0.00429741	0.24570722	0.10700702
Ba	0.51958457	0.22647579	0.32440253
Ba	0.25595474	0.24404526	0.21463051
Ba	0.74857016	0.26190887	0.98236280
Ba	0.74404520	0.75595474	0.21463051
Ba	0.23345037	0.73646704	0.00117895
Ba	0.02139456	0.75810454	0.33140802
Ba	0.50691085	0.73316520	0.11042686
Ba	0.74404520	0.24404526	0.21463051
Ba	0.22198201	0.25525376	0.99182759
Ba	0.98360781	0.21970883	0.33571234
Ba	0.50751448	0.26156039	0.11084294
Fe	0.97510134	-0.01271472	0.00968684
Fe	0.50000000	0.00000000	0.21463051
Fe	0.26415227	0.00126668	0.11299228
Fe	0.77559336	-0.07269956	0.35619439
Fe	0.96150596	0.54709259	0.98734020
Fe	0.50000000	0.50000000	0.21463051
Fe	0.25735963	0.46702457	0.10595731

Fe	0.74679401	0.51433921	0.32213556
Fe	0.75134209	0.02701196	0.10914792
Fe	0.24549451	0.04003221	0.31918603
Fe	0.00000000	0.00000000	0.21463051
Fe	0.48727950	0.00424201	0.01650533
Fe	0.76565429	0.49079005	0.09451317
Fe	0.27024004	0.47118212	0.31147542
Ce	0.00000000	0.50000000	0.21463051
Ce	0.48502172	0.49024660	0.97879020
O	0.97875204	0.76765375	0.99829336
O	0.50000000	0.75208080	0.21463051
O	0.25388051	0.77870425	0.10440793
O	0.74524658	0.70853069	0.35328020
O	0.97192140	0.13255791	0.96393037
O	0.50000000	0.24791920	0.21463051
O	0.24882237	0.24333021	0.10928917
O	0.75623398	0.34418795	0.35877527
O	0.75625855	0.71898366	0.09670489
O	0.25579690	0.69994520	0.33343829
O	0.00000000	0.77205044	0.21463051
O	0.48260902	0.80017113	0.99291350
O	0.74940460	0.25725014	0.10932989
O	0.27253366	0.26097044	0.32717026
O	0.00000000	0.22794956	0.21463051
O	0.49138652	0.18313742	0.97992420
O	0.10746449	0.01017721	0.04431560
O	0.61166381	-0.00020808	0.26277593
O	0.37604040	0.00000000	0.16141950
O	0.88827068	-0.03947248	0.39603747
O	0.14282023	0.49479643	0.05929089
O	0.62518126	0.50461351	0.28033804
O	0.37634283	0.50000000	0.16154932
O	0.85526060	0.53144559	0.94344341
O	0.87365711	0.00000000	0.16039647
O	0.36775436	-0.00758088	0.37094616
O	0.11705855	0.00140263	0.27083831
O	0.62342955	0.00955010	0.05039026
O	0.86397487	0.50000000	0.15624020
O	0.32920031	0.49916647	0.94295763
O	0.12449172	0.48564886	0.28448383

O	0.63873038	0.48401258	0.04737582
O	0.85166023	0.01202002	0.05027461
O	0.37181525	-0.00390981	0.26909112
O	0.12634283	0.00000000	0.16039647
O	0.64338645	0.00244655	0.37276524
O	0.88534888	0.46755234	0.04445338
O	0.39500800	0.49992232	0.27272565
O	0.13602522	0.50000000	0.15624020
O	0.62869123	0.50427519	0.93584902
O	0.62722376	-0.00717551	0.15768086
O	0.14395256	0.00655845	0.36919653
O	0.84396112	-0.02371902	0.29687593
O	0.35552302	0.02281827	0.05162193
O	0.62101938	0.49991344	0.17032672
O	0.08918760	0.49349688	0.96096929
O	0.86866957	0.50936317	0.28037977
O	0.37252292	0.48396507	0.05542713

S4.3: 1 oxygen vacancy, deprotonated
K-point grid: 2 2 1
79 ions

11.2817173004000004	0.0000000000000000	0.0000000000000000
0.0000000000000000	7.9773788451999996	0.0000000000000000
0.0000000000000000	0.0000000000000000	26.2817173004000004

Ba	0.00423482	0.75225824	0.10784630
Ba	0.51015356	0.76331566	0.32350565
Ba	0.25595474	0.75595474	0.21463051
Ba	0.75222818	0.74306776	0.99444594
Ba	0.00423482	0.24774176	0.10784630
Ba	0.51015356	0.23668434	0.32350565
Ba	0.25595474	0.24404526	0.21463051
Ba	0.75222818	0.25693224	0.99444594
Ba	0.74404520	0.75595474	0.21463051
Ba	0.24042433	0.73973484	0.99773849
Ba	0.00539610	0.76152551	0.33596716
Ba	0.50429161	0.73556097	0.11064544
Ba	0.74404520	0.24404526	0.21463051
Ba	0.24042433	0.26026516	0.99773849

Ba	0.00539610	0.23847449	0.33596716
Ba	0.50429161	0.26443903	0.11064544
Fe	0.99575465	0.00000000	0.01473227
Fe	0.50000000	0.00000000	0.21463051
Fe	0.26045314	0.00000000	0.11167852
Fe	0.77532322	0.00000000	0.34851587
Fe	0.98816541	0.50000000	0.99548225
Fe	0.50000000	0.50000000	0.21463051
Fe	0.25288210	0.50000000	0.10380862
Fe	0.74921480	0.50000000	0.32191717
Fe	0.74954698	0.00000000	0.10986017
Fe	0.23116134	0.00000000	0.31473797
Fe	0.00000000	0.00000000	0.21463051
Fe	0.49864281	0.00000000	0.01788169
Fe	0.76176413	0.50000000	0.09931691
Fe	0.26995764	0.50000000	0.31221286
Ce	0.00000000	0.50000000	0.21463051
Ce	0.49637616	0.50000000	0.97808221
O	0.99500604	0.80742551	0.98586332
O	0.50000000	0.75208080	0.21463051
O	0.25318602	0.75207601	0.10651867
O	0.75362810	0.68875309	0.35202251
O	0.99500604	0.19257449	0.98586332
O	0.50000000	0.24791920	0.21463051
O	0.25318602	0.24792399	0.10651867
O	0.75362810	0.31124691	0.35202251
O	0.75142063	0.74628204	0.10548037
O	0.26736676	0.70212466	0.33709914
O	0.00000000	0.77205044	0.21463051
O	0.49876794	0.80772383	0.98785508
O	0.75142063	0.25371796	0.10548037
O	0.26736676	0.29787534	0.33709914
O	0.00000000	0.22794956	0.21463051
O	0.49876794	0.19227617	0.98785508
O	0.12905204	0.00000000	0.04951944
O	0.60581363	0.00000000	0.26525838
O	0.37604040	0.00000000	0.16141950
O	0.88894889	0.00000000	0.38977585
O	0.13034183	0.50000000	0.05620521
O	0.62481637	0.50000000	0.28106897

O	0.37634283	0.50000000	0.16154932
O	0.87788532	0.50000000	0.95292274
O	0.87365711	0.00000000	0.16039647
O	0.11016701	0.00000000	0.27064238
O	0.63392431	0.00000000	0.05175520
O	0.86397487	0.50000000	0.15624020
O	0.34411000	0.50000000	0.94087003
O	0.12957647	0.50000000	0.28167321
O	0.63935078	0.50000000	0.05119344
O	0.86451591	0.00000000	0.05067486
O	0.36651946	0.00000000	0.27900961
O	0.12634283	0.00000000	0.16039647
O	0.63642540	0.00000000	0.37081006
O	0.88471680	0.50000000	0.05231568
O	0.39549570	0.50000000	0.27295605
O	0.13602522	0.50000000	0.15624020
O	0.64664552	0.50000000	0.93937564
O	0.62408950	0.00000000	0.16025528
O	0.16248533	0.00000000	0.37135487
O	0.84929679	0.00000000	0.28949837
O	0.36543754	0.00000000	0.05290946
O	0.62291491	0.50000000	0.16997048
O	0.10753907	0.50000000	0.95876190
O	0.87484025	0.50000000	0.28261934
O	0.37030259	0.50000000	0.05300301

S4.4: 1 oxygen vacancy, protonated
K-point grid: 2 2 1
81 ions

11.2817173004000004	0.0000000000000000	0.0000000000000000
0.0000000000000000	7.9773788451999996	0.0000000000000000
0.0000000000000000	0.0000000000000000	26.2817173004000004

H	0.66742877	0.00187279	0.40299571
H	0.34025924	0.46946827	0.40690304
Ba	0.00425574	0.75807843	0.10618471
Ba	0.47639936	0.75632130	0.32643468
Ba	0.25595474	0.75595474	0.21463051
Ba	0.73604030	0.74114251	0.97812060

Ba	-0.00061368	0.24692520	0.10888326
Ba	0.50736513	0.24120826	0.32495445
Ba	0.25595474	0.24404526	0.21463051
Ba	0.72038781	0.24322761	0.98541977
Ba	0.74404520	0.75595474	0.21463051
Ba	0.23569251	0.73959752	0.99555570
Ba	0.99377567	0.76764292	0.32777633
Ba	0.50556250	0.73603211	0.11092733
Ba	0.74404520	0.24404526	0.21463051
Ba	0.22424699	0.26028112	0.00053323
Ba	0.96410161	0.23572029	0.32885637
Ba	0.50790415	0.26795862	0.10991165
Fe	0.94930682	0.03187739	0.00512833
Fe	0.50000000	0.00000000	0.21463051
Fe	0.26470159	0.99855515	0.11297393
Fe	0.74041655	0.95638911	0.32143830
Fe	0.95529184	0.46702171	0.98872333
Fe	0.50000000	0.50000000	0.21463051
Fe	0.25588374	0.52827305	0.10642373
Fe	0.72756526	0.51860503	0.31244942
Fe	0.74573131	0.97225526	0.10753223
Fe	0.23418908	0.01871722	0.31325849
Fe	0.00000000	0.00000000	0.21463051
Fe	0.48734809	0.99526473	0.01864414
Fe	0.76288300	0.51276841	0.09257572
Fe	0.25600808	0.44270701	0.32072162
Ce	0.00000000	0.50000000	0.21463051
Ce	0.48121370	0.50066994	0.97931540
O	0.88133963	0.98813698	0.94956850
O	0.50000000	0.75208080	0.21463051
O	0.24710269	0.75575730	0.10914128
O	0.72659466	0.73110665	0.32965223
O	0.97483311	0.25347241	0.00631285
O	0.50000000	0.24791920	0.21463051
O	0.24594269	0.21919398	0.10311355
O	0.73553309	0.33550174	0.34603502
O	0.74345329	0.74114393	0.11033029
O	0.37435140	0.48529457	0.37326428
O	0.00000000	0.77205044	0.21463051
O	0.48844477	0.81037872	0.98385803

O	0.75666007	0.28570670	0.09364640
O	0.23488905	0.22844021	0.33615467
O	0.00000000	0.22794956	0.21463051
O	0.48024292	0.19706303	0.99322475
O	0.08949370	0.96911744	0.02451990
O	0.61811418	-0.00045092	0.27155335
O	0.37604040	0.00000000	0.16141950
O	0.84806170	0.99207872	0.36971145
O	0.14160674	0.50338740	0.05963208
O	0.59827484	0.50421097	0.27440703
O	0.37634283	0.50000000	0.16154932
O	0.85056454	0.47675334	0.94347379
O	0.87365711	0.00000000	0.16039647
O	0.10397306	0.00692312	0.27423684
O	0.62586256	0.99590866	0.05164400
O	0.86397487	0.50000000	0.15624020
O	0.32492752	0.48770347	0.94315339
O	0.12178811	0.49616846	0.28968795
O	0.63550338	0.51727819	0.04617425
O	0.85715842	0.96881668	0.05442119
O	0.36923973	0.00674704	0.27441405
O	0.12634283	0.00000000	0.16039647
O	0.61501164	0.99181010	0.37359024
O	0.88024401	0.55162523	0.04376398
O	0.36082618	0.50561438	0.27280549
O	0.13602522	0.50000000	0.15624020
O	0.61952805	0.49162716	0.93260572
O	0.62624507	0.00877751	0.15967544
O	0.23189843	0.83962233	0.34881176
O	0.86269797	0.00079235	0.27153258
O	0.35292306	0.98158958	0.05307933
O	0.61891220	0.49817385	0.16916675
O	0.07867765	0.53555887	0.96217056
O	0.86523985	0.50613170	0.27871588
O	0.37150531	0.51243079	0.05568079

S4.5: 2 oxygen vacancies, deprotonated
K-point grid: 2 2 1
78 ions

11.2817173004000004	0.0000000000000000	0.0000000000000000
0.0000000000000000	7.9773788451999996	0.0000000000000000
0.0000000000000000	0.0000000000000000	26.2817173004000004

Ba	-0.01029545	0.75384183	0.10074988
Ba	0.51444465	0.77459736	0.32123722
Ba	0.25595474	0.75595474	0.21463051
Ba	0.73990034	0.74490864	0.99005889
Ba	0.00763687	0.24279242	0.10841619
Ba	0.50441576	0.23921300	0.32251232
Ba	0.25595474	0.24404526	0.21463051
Ba	0.69777843	0.23369702	0.91561417
Ba	0.74404520	0.75595474	0.21463051
Ba	0.22258687	0.73630605	0.99685964
Ba	0.01849965	0.75422986	0.33650146
Ba	0.51251193	0.73778299	0.11010397
Ba	0.74404520	0.24404526	0.21463051
Ba	0.23366069	0.24745197	0.00189378
Ba	0.02543216	0.22451364	0.33671094
Ba	0.49763037	0.26563381	0.11053252
Fe	0.93839890	0.06627531	0.00192051
Fe	0.50000000	0.00000000	0.21463051
Fe	0.26629927	-0.00691457	0.11359225
Fe	0.78428481	-0.03995624	0.34851181
Fe	0.93060149	0.46452772	0.99257822
Fe	0.50000000	0.50000000	0.21463051
Fe	0.25468666	0.53291309	0.10604484
Fe	0.75873943	0.55526355	0.31644346
Fe	0.74139943	0.03604447	0.10618868
Fe	0.24148132	-0.04621096	0.31550372
Fe	0.00000000	0.00000000	0.21463051
Fe	0.49662988	0.00367688	0.01635240
Fe	0.75196126	0.48626899	0.09654566
Fe	0.27620838	0.51609353	0.31362898
Ce	0.00000000	0.50000000	0.21463051
Ce	0.48505175	0.49970627	0.98266905
O	0.85247204	0.01640681	0.95056236
O	0.50000000	0.75208080	0.21463051
O	0.24364101	0.76012147	0.10521120
O	0.76370695	0.73381575	0.35258360

O	-0.00048888	0.26767682	0.99011746
O	0.50000000	0.24791920	0.21463051
O	0.24644729	0.21365315	0.10377040
O	0.74967605	0.71436109	0.09794831
O	0.27814004	0.73561292	0.32940174
O	0.00000000	0.77205044	0.21463051
O	0.49630801	0.80549695	0.98836032
O	0.73811956	0.25998934	0.11230005
O	0.27275506	0.33558511	0.34786566
O	0.00000000	0.22794956	0.21463051
O	0.49601780	0.18510170	0.97927007
O	0.07688545	-0.01481900	0.01974594
O	0.60928310	0.00191383	0.26409509
O	0.37604040	0.00000000	0.16141950
O	0.89793328	-0.00344918	0.38948932
O	0.14363039	0.49490964	0.05849068
O	0.63414348	0.49287176	0.28375223
O	0.37634283	0.50000000	0.16154932
O	0.83560475	0.45874936	0.94320384
O	0.87365711	0.00000000	0.16039647
O	0.12070152	0.00531482	0.27161662
O	0.62690036	0.00006152	0.05361786
O	0.86397487	0.50000000	0.15624020
O	0.32067521	0.49159406	0.94820962
O	0.14041533	0.50758225	0.27823893
O	0.62907818	0.48880961	0.04814116
O	0.85768779	0.00690804	0.05633790
O	0.37136369	0.00727509	0.27610222
O	0.12634283	0.00000000	0.16039647
O	0.64916771	0.03160635	0.36703254
O	0.87513842	0.46097600	0.05507467
O	0.40128195	0.50402484	0.27344098
O	0.13602522	0.50000000	0.15624020
O	0.60302501	0.51278689	0.92472456
O	0.62601250	-0.00697869	0.15998987
O	0.17241308	-0.00478180	0.37173321
O	0.85770440	-0.00066636	0.28990073
O	0.35979146	-0.00865439	0.05090105
O	0.62215481	0.50106680	0.17011477
O	-0.00591296	0.65542073	0.98944749

O	0.89385512	0.47734370	0.29696941
O	0.37195300	0.50988378	0.05697342

S4.6: 2 oxygen vacancies, protonated

K-point grid: 2 2 1

80 ions

11.2817173004000004	0.0000000000000000	0.0000000000000000
0.0000000000000000	7.9773788451999996	0.0000000000000000
0.0000000000000000	0.0000000000000000	26.2817173004000004

H	0.17797617	0.96502031	0.40534086
H	0.64363004	0.47472278	0.40828194
Ba	0.99562175	0.75343065	0.10104817
Ba	0.51851207	0.76565697	0.32306047
Ba	0.25595474	0.75595474	0.21463051
Ba	0.73733795	0.74808728	0.99133962
Ba	0.99951657	0.24270840	0.10822828
Ba	0.49459997	0.24495473	0.32271893
Ba	0.25595474	0.24404526	0.21463051
Ba	0.70252879	0.24124590	0.91396384
Ba	0.74404520	0.75595474	0.21463051
Ba	0.22720410	0.74056846	0.99991896
Ba	0.99073111	0.75825439	0.32832675
Ba	0.50483142	0.73503004	0.10986323
Ba	0.74404520	0.24404526	0.21463051
Ba	0.22847136	0.25287998	0.99818602
Ba	0.02752788	0.25127549	0.33191504
Ba	0.50496348	0.26231341	0.11113097
Fe	0.93685527	0.06655606	0.00162438
Fe	0.50000000	0.00000000	0.21463051
Fe	0.26707553	0.00316316	0.11316100
Fe	0.75689857	0.08824548	0.35446472
Fe	0.93014650	0.47054858	0.99294514
Fe	0.50000000	0.50000000	0.21463051
Fe	0.25434745	0.46730409	0.10602132
Fe	0.74005764	0.46547736	0.32505298
Fe	0.74167740	0.03158388	0.10647252
Fe	0.23525293	0.94642765	0.31475153
Fe	0.00000000	0.00000000	0.21463051

Fe	0.49737418	0.00748725	0.01624090
Fe	0.75068720	0.49030522	0.09740363
Fe	0.27136430	0.52355498	0.31651141
Ce	0.00000000	0.50000000	0.21463051
Ce	0.48550848	0.49552937	0.98294347
O	0.85036997	0.01744678	0.95033632
O	0.50000000	0.75208080	0.21463051
O	0.24619517	0.78628164	0.10087226
O	0.61438054	0.50464826	0.37465496
O	0.99443708	0.27021691	0.98997128
O	0.50000000	0.24791920	0.21463051
O	0.24235533	0.23926941	0.10848303
O	0.74789296	0.71771138	0.09952492
O	0.27009839	0.73865447	0.33550697
O	0.00000000	0.77205044	0.21463051
O	0.48770789	0.80164092	0.99352974
O	0.74155436	0.25762678	0.11223424
O	0.26158524	0.33827503	0.34976837
O	0.00000000	0.22794956	0.21463051
O	0.50519784	0.18021229	0.97535373
O	0.07658933	0.98920229	0.01855701
O	0.60731941	0.00248453	0.26530159
O	0.37604040	0.00000000	0.16141950
O	0.79279616	0.29394156	0.36186611
O	0.14220019	0.49252530	0.05830234
O	0.63318350	0.50702668	0.27629644
O	0.37634283	0.50000000	0.16154932
O	0.83750976	0.46900837	0.94264333
O	0.87365711	0.00000000	0.16039647
O	0.10662467	0.00477356	0.27425476
O	0.62652116	0.99837019	0.05415157
O	0.86397487	0.50000000	0.15624020
O	0.32137898	0.49501427	0.94786009
O	0.13396281	0.51594279	0.28082202
O	0.62856418	0.49073867	0.04871200
O	0.85786539	0.00232564	0.05616616
O	0.36182659	0.00289382	0.27702828
O	0.12634283	0.00000000	0.16039647
O	0.63127768	0.01228023	0.37857911
O	0.87316953	0.46739240	0.05522415

O	0.39179472	0.50604200	0.27367046
O	0.13602522	0.50000000	0.15624020
O	0.60350574	0.51768921	0.92527581
O	0.62499904	0.99050981	0.16026019
O	0.13075438	0.98127437	0.37483144
O	0.85158889	0.00284723	0.31080335
O	0.36163253	0.02727652	0.05123724
O	0.62120203	0.50090052	0.16844398
O	0.99743035	0.65847274	0.99058011
O	0.87173779	0.49686456	0.28655840
O	0.37097718	0.48092083	0.05646456

S4.7: 3 oxygen vacancies, deprotonated
K-point grid: 2 2 1
77 ions

11.2817173004000004	0.0000000000000000	0.0000000000000000
0.0000000000000000	7.9773788451999996	0.0000000000000000
0.0000000000000000	0.0000000000000000	26.2817173004000004

Ba	0.98863874	0.75435876	0.10077033
Ba	0.51876664	0.77081592	0.32025028
Ba	0.25595474	0.75595474	0.21463051
Ba	0.73821013	0.74667858	0.99008523
Ba	0.00590430	0.24289968	0.10846673
Ba	0.49809244	0.23707985	0.32279392
Ba	0.25595474	0.24404526	0.21463051
Ba	0.69698575	0.23577029	0.91594184
Ba	0.74404520	0.75595474	0.21463051
Ba	0.22175218	0.73755494	0.99711408
Ba	0.00070557	0.77507859	0.33222071
Ba	0.51070056	0.73756507	0.11008079
Ba	0.74404520	0.24404526	0.21463051
Ba	0.23227728	0.24862647	0.00200557
Ba	0.03940559	0.24576099	0.33907740
Ba	0.49660790	0.26595472	0.11060220
Fe	0.93741206	0.06742115	0.00180015
Fe	0.50000000	0.00000000	0.21463051
Fe	0.26439893	0.99344166	0.11401710
Fe	0.77918440	0.01888178	0.32943991

Fe	0.92909819	0.46640635	0.99289680
Fe	0.50000000	0.50000000	0.21463051
Fe	0.25366701	0.53064166	0.10594667
Fe	0.74234119	0.49619463	0.30340030
Fe	0.73955492	0.03634694	0.10585996
Fe	0.24022117	0.94935250	0.31688242
Fe	0.00000000	0.00000000	0.21463051
Fe	0.49452749	0.00432851	0.01669805
Fe	0.74954295	0.48686606	0.09676360
Fe	0.27247431	0.52212290	0.31674325
Ce	0.00000000	0.50000000	0.21463051
Ce	0.48366276	0.50023962	0.98269441
O	0.85153175	0.01787125	0.95039385
O	0.50000000	0.75208080	0.21463051
O	0.24388229	0.75887874	0.10541501
O	0.99787009	0.26941347	0.99027291
O	0.50000000	0.24791920	0.21463051
O	0.24487849	0.21483269	0.10408989
O	0.74879542	0.71485122	0.09762595
O	0.28188441	0.73787591	0.33143978
O	0.00000000	0.77205044	0.21463051
O	0.49371687	0.80576439	0.98896702
O	0.73731857	0.25957784	0.11176392
O	0.27072802	0.35314328	0.35597238
O	0.00000000	0.22794956	0.21463051
O	0.49418116	0.18536585	0.97947769
O	0.07583277	0.98656592	0.01949209
O	0.61291335	0.00288173	0.26476192
O	0.37604040	0.00000000	0.16141950
O	0.88706541	0.02694182	0.37429311
O	0.14209854	0.49634271	0.05836997
O	0.61465594	0.49602420	0.27038192
O	0.37634283	0.50000000	0.16154932
O	0.83488166	0.46119390	0.94324158
O	0.87365711	0.00000000	0.16039647
O	0.12422765	0.01124517	0.27219466
O	0.62592546	0.00054135	0.05320874
O	0.86397487	0.50000000	0.15624020
O	0.31937362	0.49281426	0.94814083
O	0.13951949	0.51128372	0.27953990

O	0.62798374	0.48936292	0.04774509
O	0.85667179	0.00716778	0.05614635
O	0.37126582	0.00599437	0.27783445
O	0.12634283	0.00000000	0.16039647
O	0.64958085	0.01490662	0.36155354
O	0.87329377	0.46181111	0.05528401
O	0.39108115	0.49763891	0.27416548
O	0.13602522	0.50000000	0.15624020
O	0.60172336	0.51446282	0.92467461
O	0.62603758	0.99191131	0.15956923
O	0.16742209	0.99363000	0.37211357
O	0.86683700	0.99845912	0.27361929
O	0.35753466	0.99262444	0.05121148
O	0.61993348	0.50110716	0.16679623
O	0.99265069	0.65727323	0.99002031
O	0.89192515	0.49321523	0.30485158
O	0.37055675	0.50811067	0.05675783

S4.8: 3 oxygen vacancies, protonated

K-point grid: 2 2 1

79 ions

11.2817173004000004	0.0000000000000000	0.0000000000000000
0.0000000000000000	7.9773788451999996	0.0000000000000000
0.0000000000000000	0.0000000000000000	26.2817173004000004

H	0.68167764	0.00433869	0.39789756
H	0.24036963	0.24408718	0.36956615
Ba	0.98817968	0.75430035	0.10071876
Ba	0.50471429	0.76502949	0.32078806
Ba	0.25595474	0.75595474	0.21463051
Ba	0.73728621	0.74671391	0.99025214
Ba	0.00570969	0.24302126	0.10840972
Ba	0.49522826	0.24314657	0.32301645
Ba	0.25595474	0.24404526	0.21463051
Ba	0.69583871	0.23576075	0.91627730
Ba	0.74404520	0.75595474	0.21463051
Ba	0.22066500	0.73777165	0.99718912
Ba	-0.00632856	0.77605906	0.33024105
Ba	0.51024136	0.73788200	0.11027503

Ba	0.74404520	0.24404526	0.21463051
Ba	0.23133295	0.24910512	0.00205535
Ba	-0.00234094	0.22666333	0.32934288
Ba	0.49610392	0.26599124	0.11046926
Fe	0.93638686	0.06756546	0.00191250
Fe	0.50000000	0.00000000	0.21463051
Fe	0.26296479	0.99352749	0.11344970
Fe	0.75032723	0.00304696	0.31572422
Fe	0.92832454	0.46658646	0.99282329
Fe	0.50000000	0.50000000	0.21463051
Fe	0.25344665	0.53025574	0.10590481
Fe	0.73265535	0.50173428	0.30344016
Fe	0.73938680	0.03626281	0.10608386
Fe	0.23771875	0.96280310	0.31672500
Fe	0.00000000	0.00000000	0.21463051
Fe	0.49315779	0.00417971	0.01682065
Fe	0.74918987	0.48674265	0.09663144
Fe	0.26127593	0.52736783	0.32032617
Ce	0.00000000	0.50000000	0.21463051
Ce	0.48261281	0.50068856	0.98259546
O	0.85045536	0.01778313	0.95051141
O	0.50000000	0.75208080	0.21463051
O	0.24401821	0.75804474	0.10606157
O	0.99685215	0.26937860	0.99003111
O	0.50000000	0.24791920	0.21463051
O	0.24460600	0.21584509	0.10409434
O	0.74828911	0.71488504	0.09780405
O	0.25834572	0.74562196	0.33385999
O	0.00000000	0.77205044	0.21463051
O	0.49289186	0.80581318	0.98895300
O	0.73671265	0.25975730	0.11190611
O	0.28556108	0.35389837	0.36528449
O	0.00000000	0.22794956	0.21463051
O	0.49325313	0.18581770	0.97977967
O	0.07472385	0.98713332	0.01998322
O	0.62167584	0.00174659	0.27087013
O	0.37604040	0.00000000	0.16141950
O	0.84971167	-0.00018923	0.36707745
O	0.14160915	0.49689585	0.05832218
O	0.60621061	0.49761886	0.26996036

O	0.37634283	0.50000000	0.16154932
O	0.83331054	0.46168806	0.94339308
O	0.87365711	0.00000000	0.16039647
O	0.12172201	0.00796691	0.27090280
O	0.62337985	0.00037205	0.05419045
O	0.86397487	0.50000000	0.15624020
O	0.31815079	0.49323754	0.94811303
O	0.12935661	0.49138182	0.28393827
O	0.62763601	0.48947657	0.04773902
O	0.85516709	0.00752620	0.05615640
O	0.37144529	0.00307248	0.27812238
O	0.12634283	0.00000000	0.16039647
O	0.62725125	0.00552836	0.36890122
O	0.87291542	0.46172411	0.05524511
O	0.38007683	0.50211882	0.27639322
O	0.13602522	0.50000000	0.15624020
O	0.60025632	0.51456584	0.92444456
O	0.62733216	0.99331301	0.16134331
O	0.17913222	0.06013399	0.36995582
O	0.87374442	-0.00155128	0.26908383
O	0.35567170	0.99201151	0.05107810
O	0.62013458	0.50072275	0.16711106
O	0.99160214	0.65766975	0.98988786
O	0.88308370	0.50456081	0.30197540
O	0.37033149	0.50871731	0.05664201

S5: Orthorhombic BCF8515 Free Energy Equations

T is temperature in K

S5.1.1: ΔG for rxn(I) from oxygen defect free surface

ΔG = -6.24151*10^18*T*(1.380000000*10^(-23)*ln(0.01007824655*exp(-6335.270153/T)*T*(1 - exp(-5283.946913/T))*(1 - exp(-4995.667899/T))*(1 - exp(-996.9201116/T))*(1 - exp(-747.3455797/T))*(1 - exp(-384.3152022/T))*(1 - exp(-254.5651583/T))/((1 - exp(-6335.270153/T))*exp(-5283.946913/T)*exp(-4995.667899/T)*exp(-996.9201116/T)*exp(-747.3455797/T)*exp(-384.3152022/T)*exp(-254.5651583/T))) + 1.380000000*10^(-23)*ln(3.631578947*10^(-26)*T) + 2.070000000*10^(-23)*ln(6.610384564*10^17*T) + 3.450000000*10^(-23)) + 3.574774920

S5.1.2: ΔG for rxn(II) from oxygen defect free surface

ΔG = -6.24151*10^18*T*(1.380000000*10^(-23)*ln(0.01007824655*exp(-6335.270153/T)*T*(1 - exp(-4937.378304/T))*(1 - exp(-4192.097348/T))*(1 - exp(-1353.720609/T))*(1 - exp(-1122.314761/T))*(1 - exp(-1051.805478/T))*(1 - exp(-801.0065768/T))/((1 - exp(-6335.270153/T))*exp(-

4937.378304/T)*exp(-4192.097348/T)*exp(-1353.720609/T)*exp(-1122.314761/T)*exp(-1051.805478/T)*exp(-801.0065768/T))) + 1.380000000*10^(-23)*ln(3.631578947*10^(-26)*T) + 2.070000000*10^(-23)*ln(6.610384564*10^17*T) + 3.450000000*10^(-23)) + 1.333386264

S5.1.3: ΔG for rxn(III) from oxygen defect free surface

ΔG = -6.24151*10^18*T*(1.380000000*10^(-23)*ln(0.6526881536*sqrt(exp(-2274.585976/T)*T/(1 - exp(-2274.585976/T)))*(1 - exp(-1454.603859/T))*(1 - exp(-222.6772932/T))*(1 - exp(-121.4200096/T))/(exp(-1454.603859/T)*exp(-222.6772932/T)*exp(-121.4200096/T))) + 6.900000000*10^(-24)*ln(3.631578947*10^(-26)*T) + 1.035000000*10^(-23)*ln(1.049368400*10^19*T) + 1.725000000*10^(-23)) + 11.99805467

S5.2.1: ΔG for rxn(I) from 1 oxygen defect surface

ΔG = -6.24151*10^18*T*(1.380000000*10^(-23)*ln(0.01007824655*exp(-6335.270153/T)*T*(1 - exp(-4937.378304/T))*(1 - exp(-4192.097348/T))*(1 - exp(-1353.720609/T))*(1 - exp(-1122.314761/T))*(1 - exp(-1051.805478/T))*(1 - exp(-801.0065768/T))/((1 - exp(-6335.270153/T))*exp(-4937.378304/T)*exp(-4192.097348/T)*exp(-1353.720609/T)*exp(-1122.314761/T)*exp(-1051.805478/T)*exp(-801.0065768/T))) + 1.380000000*10^(-23)*ln(3.631578947*10^(-26)*T) + 2.070000000*10^(-23)*ln(6.610384564*10^17*T) + 3.450000000*10^(-23)) + 1.333386264

S5.2.2: ΔG for rxn(II) from 1 oxygen defect surface

ΔG = -6.24151*10^18*T*(1.380000000*10^(-23)*ln(0.008696458588*exp(-5509.855101/T)*exp(-5346.461783/T)*exp(-2278.729414/T)*T^(3/2)*(1 - exp(-4938.962783/T))*(1 - exp(-4322.461497/T))*(1 - exp(-1363.035420/T))*(1 - exp(-1132.551451/T))*(1 - exp(-1051.402157/T))*(1 - exp(-800.3055652/T))*(1 - exp(-653.2467768/T))*(1 - exp(-216.4488532/T))*(1 - exp(-193.2525716/T))/((1 - exp(-5509.855101/T))*(1 - exp(-5346.461783/T))*(1 - exp(-2278.729414/T))*exp(-4938.962783/T)*exp(-4322.461497/T)*exp(-1363.035420/T)*exp(-1132.551451/T)*exp(-1051.402157/T)*exp(-800.3055652/T)*exp(-653.2467768/T)*exp(-216.4488532/T)*exp(-193.2525716/T))) + 1.380000000*10^(-23)*ln(3.631578947*10^(-26)*T) + 2.070000000*10^(-23)*ln(6.568918916*10^18*T) + 3.450000000*10^(-23)) + 1.111094886

S5.2.3: ΔG for rxn(III) from 1 oxygen defect surface

ΔG = -6.24151*10^18*T*(1.380000000*10^(-23)*ln(0.6526881536*sqrt(exp(-2274.585976/T)*T/(1 - exp(-2274.585976/T)))*(1 - exp(-1235.100804/T))*(1 - exp(-310.9697035/T))*(1 - exp(-247.8071184/T))/(exp(-1235.100804/T)*exp(-310.9697035/T)*exp(-247.8071184/T))) + 6.900000000*10^(-24)*ln(3.631578947*10^(-26)*T) + 1.035000000*10^(-23)*ln(1.049368400*10^19*T) + 1.725000000*10^(-23)) + 10.50302578

S5.3.1: ΔG for rxn(I) from 2 oxygen defect surface

ΔG = -6.24151*10^18*T*(1.380000000*10^(-23)*ln(0.01007824655*exp(-6335.270153/T)*T*(1 - exp(-6236.122319/T))*(1 - exp(-4472.309928/T))*(1 - exp(-1343.608757/T))*(1 - exp(-1009.783194/T))*(1 -

exp(-941.7562609/T))*(1 - exp(-370.0289700/T))/((1 - exp(-6335.270153/T))*exp(-6236.122319/T)*exp(-4472.309928/T)*exp(-1343.608757/T)*exp(-1009.783194/T)*exp(-941.7562609/T)*exp(-370.0289700/T))) + 1.380000000*10^(-23)*ln(3.631578947*10^(-26)*T) + 2.070000000*10^(-23)*ln(6.610384564*10^17*T) + 3.450000000*10^(-23)) + 0.5755102884

S.5.3.2: ΔG for rxn(II) from 2 oxygen defect surface

ΔG = -6.24151*10^18*T*(1.380000000*10^(-23)*ln(0.008696458588*exp(-5509.855101/T)*exp(-5346.461783/T)*exp(-2278.729414/T)*T^(3/2)*(1 - exp(-5041.137623/T))*(1 - exp(-4543.371377/T))*(1 - exp(-1306.642399/T))*(1 - exp(-1034.923583/T))*(1 - exp(-980.7584333/T))*(1 - exp(-934.6021014/T))*(1 - exp(-428.7665394/T))*(1 - exp(-328.6731271/T))*(1 - exp(-266.0761528/T))/((1 - exp(-5509.855101/T))*(1 - exp(-5346.461783/T))*(1 - exp(-2278.729414/T))*exp(-5041.137623/T)*exp(-4543.371377/T)*exp(-1306.642399/T)*exp(-1034.923583/T)*exp(-980.7584333/T)*exp(-934.6021014/T)*exp(-428.7665394/T)*exp(-328.6731271/T)*exp(-266.0761528/T))) + 1.380000000*10^(-23)*ln(3.631578947*10^(-26)*T) + 2.070000000*10^(-23)*ln(6.568918916*10^18*T) + 3.450000000*10^(-23)) + 1.567143297

S.5.3.3: ΔG for rxn(III) from 2 oxygen defect surface

ΔG = -6.24151*10^18*T*(1.380000000*10^(-23)*ln(0.6526881536*sqrt(exp(-2274.585976/T)*T/(1 - exp(-2274.585976/T)))*(1 - exp(-1303.694309/T))*(1 - exp(-353.2186159/T))*(1 - exp(-292.1057696/T))/(exp(-1303.694309/T)*exp(-353.2186159/T)*exp(-292.1057696/T))) + 6.900000000*10^(-24)*ln(3.631578947*10^(-26)*T) + 1.035000000*10^(-23)*ln(1.049368400*10^19*T) + 1.725000000*10^(-23)) + 5.558620149

S6: Cubic BCF8515 Free Energy Equations

T is temperature in K

S6.1.1: ΔG for rxn(I) from oxygen defect free surface

ΔG =-6.24151*10^18*T*(1.380000000*10^(-23)*ln(0.01007824655*exp(-6335.270153/T)*T*(1 - exp(-487.0398087/T))*(1 - exp(-488.4678078/T))*(1 - exp(-675.6321417/T))*(1 - exp(-677.2298719/T))*(1 - exp(-5165.883404/T))*(1 - exp(-5249.052333/T))/((1 - exp(-6335.270153/T))*exp(-487.0398087/T)*exp(-488.4678078/T)*exp(-675.6321417/T)*exp(-677.2298719/T)*exp(-5165.883404/T)*exp(-5249.052333/T))) + 1.380000000*10^(-23)*ln(3.631578947*10^(-26)*T) + 2.070000000*10^(-23)*ln(6.610384564*10^17*T) + 3.450000000*10^(-23)) + 2.034207973

S6.1.2: ΔG for rxn(II) from oxygen defect free surface

ΔG =-6.24151*10^18*T*(1.380000000*10^(-23)*ln(0.008696458588*exp(-5509.855101/T)*exp(-5346.461783/T)*exp(-2278.729414/T)*T^(3/2)*(1 - exp(-102.8556380/T))*(1 - exp(-106.9295238/T))*(1 - exp(-383.2334837/T))*(1 - exp(-488.4366463/T))*(1 - exp(-491.2900036/T))*(1 - exp(-676.7502551/T))*(1 - exp(-680.3466367/T))*(1 - exp(-5166.231606/T))*(1 - exp(-5409.609867/T))/((1 - exp(-5509.855101/T))*(1 - exp(-5346.461783/T))*(1 - exp(-2278.729414/T))*exp(-102.8556380/T)*exp(-106.9295238/T)*exp(-383.2334837/T)*exp(-

488.4366463/T)*exp(-491.2900036/T)*exp(-676.7502551/T)*exp(-680.3466367/T)*exp(-5166.231606/T)*exp(-5409.609867/T))) + 1.380000000*10^(-23)*ln(3.631578947*10^(-26)*T) + 2.070000000*10^(-23)*ln(6.568918916*10^18*T) + 3.450000000*10^(-23)) + 1.479200421

S6.1.1: ΔG for rxn(III) from oxygen defect free surface

ΔG = -6.24151*10^18*T*(1.380000000*10^(-23)*ln(0.6526881536*sqrt(exp(-2274.585976/T)*T/(1 - exp(-2274.585976/T)))*(1 - exp(-1454.603859/T))*(1 - exp(-222.6772932/T))*(1 - exp(-121.4200096/T))/(exp(-1454.603859/T)*exp(-222.6772932/T)*exp(-121.4200096/T))) + 6.900000000*10^(-24)*ln(3.631578947*10^(-26)*T) + 1.035000000*10^(-23)*ln(1.049368400*10^19*T) + 1.725000000*10^(-23)) + 3.873044200

S6.2.1: ΔG for rxn(I) from 1 oxygen defect surface

ΔG = -6.24151*10^18*T*(1.380000000*10^(-23)*ln(0.01007824655*exp(-6335.270153/T)*T*(1 - exp(-538.3626920/T))*(1 - exp(-652.3645105/T))*(1 - exp(-670.5889394/T))*(1 - exp(-772.5057978/T))*(1 - exp(-5098.441143/T))*(1 - exp(-5128.849634/T))/((1 - exp(-6335.270153/T))*exp(-538.3626920/T)*exp(-652.3645105/T)*exp(-670.5889394/T)*exp(-772.5057978/T)*exp(-5098.441143/T)*exp(-5128.849634/T))) + 1.380000000*10^(-23)*ln(3.631578947*10^(-26)*T) + 2.070000000*10^(-23)*ln(6.610384564*10^17*T) + 3.450000000*10^(-23)) + 0.2681870741

S6.2.2: ΔG for rxn(II) from 1 oxygen defect surface

ΔG = -6.24151*10^18*T*(1.380000000*10^(-23)*ln(0.008696458588*exp(-5509.855101/T)*exp(-5346.461783/T)*exp(-2278.729414/T)*T^(3/2)*(1 - exp(-213.4512604/T))*(1 - exp(-233.5029768/T))*(1 - exp(-336.2794870/T))*(1 - exp(-564.0609128/T))*(1 - exp(-648.8068286/T))*(1 - exp(-668.6268751/T))*(1 - exp(-785.0251449/T))*(1 - exp(-5097.718717/T))*(1 - exp(-5283.596455/T))/((1 - exp(-5509.855101/T))*(1 - exp(-5346.461783/T))*(1 - exp(-2278.729414/T))*exp(-213.4512604/T)*exp(-233.5029768/T)*exp(-336.2794870/T)*exp(-564.0609128/T)*exp(-648.8068286/T)*exp(-668.6268751/T)*exp(-785.0251449/T)*exp(-5097.718717/T)*exp(-5283.596455/T))) + 1.380000000*10^(-23)*ln(3.631578947*10^(-26)*T) + 2.070000000*10^(-23)*ln(6.568918916*10^18*T) + 3.450000000*10^(-23)) + 1.599230900

S6.2.3: ΔG for rxn(III) from 1 oxygen defect surface

ΔG = -6.24151*10^18*T*(1.380000000*10^(-23)*ln(0.6526881536*sqrt(exp(-2274.585976/T)*T/(1 - exp(-2274.585976/T)))*(1 - exp(-1235.100804/T))*(1 - exp(-310.9697035/T))*(1 - exp(-247.8071184/T))/(exp(-1235.100804/T)*exp(-310.9697035/T)*exp(-247.8071184/T))) + 6.900000000*10^(-24)*ln(3.631578947*10^(-26)*T) + 1.035000000*10^(-23)*ln(1.049368400*10^19*T) + 1.725000000*10^(-23)) + 3.135890662

S7: Cubic BCF1585 Free Energy Equations

T is temperature in K

S7.1.1: ΔG for rxn(I) from oxygen defect free surface

ΔG = -6.24151*10^18*T*(1.380000000*10^(-23)*ln(0.01007824655*exp(-6335.270153/T)*T*(1 - exp(-5112.775246/T))*(1 - exp(-5020.587420/T))*(1 - exp(-1078.059803/T))*(1 - exp(-1057.874510/T))*(1 - exp(-818.3734188/T))*(1 - exp(-717.6678217/T))/((1 - exp(-6335.270153/T))*exp(-5112.775246/T)*exp(-5020.587420/T)*exp(-1078.059803/T)*exp(-1057.874510/T)*exp(-818.3734188/T)*exp(-717.6678217/T))) + 1.380000000*10^(-23)*ln(3.631578947*10^(-26)*T) + 2.070000000*10^(-23)*ln(6.610384564*10^17*T) + 3.450000000*10^(-23)) + 3.634855696

S7.1.2: ΔG for rxn(II) from oxygen defect free surface

ΔG = -6.24151*10^18*T*(1.380000000*10^(-23)*ln(0.008696458588*exp(-5509.855101/T)*exp(-5346.461783/T)*exp(-2278.729414/T)*T^(3/2)*(1 - exp(-5263.684797/T))*(1 - exp(-5021.307638/T))*(1 - exp(-1116.917932/T))*(1 - exp(-1058.902020/T))*(1 - exp(-855.6470696/T))*(1 - exp(-726.1615855/T))*(1 - exp(-537.1045203/T))*(1 - exp(-432.2115793/T))*(1 - exp(-307.6451800/T))/((1 - exp(-5509.855101/T))*(1 - exp(-5346.461783/T))*(1 - exp(-2278.729414/T))*exp(-5263.684797/T)*exp(-5021.307638/T)*exp(-1116.917932/T)*exp(-1058.902020/T)*exp(-855.6470696/T)*exp(-726.1615855/T)*exp(-537.1045203/T)*exp(-432.2115793/T)*exp(-307.6451800/T))) + 1.380000000*10^(-23)*ln(3.631578947*10^(-26)*T) + 2.070000000*10^(-23)*ln(6.568918916*10^18*T) + 3.450000000*10^(-23)) + 2.163045223

S7.1.3: ΔG for rxn(III) from oxygen defect free surface

ΔG = -6.24151*10^18*T*(1.380000000*10^(-23)*ln(0.6526881536*sqrt(exp(-2274.585976/T)*T/(1 - exp(-2274.585976/T)))*(1 - exp(-1352.078513/T))*(1 - exp(-584.6484710/T))*(1 - exp(-382.7047961/T))/(exp(-1352.078513/T)*exp(-584.6484710/T)*exp(-382.7047961/T))) + 6.900000000*10^(-24)*ln(3.631578947*10^(-26)*T) + 1.035000000*10^(-23)*ln(1.049368400*10^19*T) + 1.725000000*10^(-23)) + 0.6836825224

S7.2.1: ΔG for rxn(I) from 1 oxygen defect surface

ΔG = -6.24151*10^18*T*(1.380000000*10^(-23)*ln(0.01007824655*exp(-6335.270153/T)*T*(1 - exp(-5235.020145/T))*(1 - exp(-5134.285739/T))*(1 - exp(-1063.007259/T))*(1 - exp(-1054.273423/T))*(1 - exp(-749.9047522/T))*(1 - exp(-534.7037957/T))/((1 - exp(-6335.270153/T))*exp(-5235.020145/T)*exp(-5134.285739/T)*exp(-1063.007259/T)*exp(-1054.273423/T)*exp(-749.9047522/T)*exp(-534.7037957/T))) + 1.380000000*10^(-23)*ln(3.631578947*10^(-26)*T) + 2.070000000*10^(-23)*ln(6.610384564*10^17*T) + 3.450000000*10^(-23)) + 4.079675630

S7.2.2: ΔG for rxn(II) from 1 oxygen defect surface

ΔG = -6.24151*10^18*T*(1.380000000*10^(-23)*ln(0.008696458588*exp(-5509.855101/T)*exp(-5346.461783/T)*exp(-2278.729414/T)*T^(3/2)*(1 - exp(-5287.548000/T))*(1 - exp(-5234.395957/T))*(1 - exp(-1105.418461/T))*(1 - exp(-1056.414870/T))*(1 - exp(-774.1712768/T))*(1 -

exp(-535.6736884/T))*(1 - exp(-518.8974246/T))*(1 - exp(-429.9697826/T))*(1 - exp(-
334.5438591/T))/((1 - exp(-5509.855101/T))*(1 - exp(-5346.461783/T))*(1 - exp(-
2278.729414/T))*exp(-5287.548000/T)*exp(-5234.395957/T)*exp(-1105.418461/T)*exp(-
1056.414870/T)*exp(-774.1712768/T)*exp(-535.6736884/T)*exp(-518.8974246/T)*exp(-
429.9697826/T)*exp(-334.5438591/T))) + 1.380000000*10^(-23)*ln(3.631578947*10^(-26)*T) +
2.070000000*10^(-23)*ln(6.568918916*10^18*T) + 3.450000000*10^(-23)) + 1.636823514

S7.2.3: ΔG for rxn(III) from 1 oxygen defect surface

ΔG = -6.24151*10^18*T*(1.380000000*10^(-23)*ln(0.6526881536*sqrt(exp(-2274.585976/T)*T/(1 -
exp(-2274.585976/T)))*(1 - exp(-1355.405917/T))*(1 - exp(-546.6546029/T))*(1 - exp(-
399.2501101/T))/(exp(-1355.405917/T)*exp(-546.6546029/T)*exp(-399.2501101/T))) +
6.900000000*10^(-24)*ln(3.631578947*10^(-26)*T) + 1.035000000*10^(-23)*ln(1.049368400*10^19*T)
+ 1.725000000*10^(-23)) + 1.935105277

S7.3.1: ΔG for rxn(I) from 2 oxygen defect surface

ΔG = -6.24151*10^18*T*(1.380000000*10^(-23)*ln(0.01007824655*exp(-6335.270153/T)*T*(1 - exp(-
5253.553739/T))*(1 - exp(-5220.327710/T))*(1 - exp(-1067.904738/T))*(1 - exp(-1025.805630/T))*(1 -
exp(-672.0156420/T))*(1 - exp(-544.9596913/T))/((1 - exp(-6335.270153/T))*exp(-
5253.553739/T)*exp(-5220.327710/T)*exp(-1067.904738/T)*exp(-1025.805630/T)*exp(-
672.0156420/T)*exp(-544.9596913/T))) + 1.380000000*10^(-23)*ln(3.631578947*10^(-26)*T) +
2.070000000*10^(-23)*ln(6.610384564*10^17*T) + 3.450000000*10^(-23)) + 2.302024926

S7.3.2: ΔG for rxn(II) from 2 oxygen defect surface

ΔG = 6.24151*10^18*T*(1.380000000*10^(-23)*ln(0.008696458588*exp(-5509.855101/T)*exp(-
5346.461783/T)*exp(-2278.729414/T)*T^(3/2)*(1 - exp(-5380.408029/T))*(1 - exp(-
5253.265652/T))*(1 - exp(-1115.904826/T))*(1 - exp(-1025.455125/T))*(1 - exp(-700.9683812/T))*(1 -
exp(-569.5527145/T))*(1 - exp(-544.9068754/T))*(1 - exp(-392.7811175/T))*(1 - exp(-
269.3867520/T))/((1 - exp(-5509.855101/T))*(1 - exp(-5346.461783/T))*(1 - exp(-
2278.729414/T))*exp(-5380.408029/T)*exp(-5253.265652/T)*exp(-1115.904826/T)*exp(-
1025.455125/T)*exp(-700.9683812/T)*exp(-569.5527145/T)*exp(-544.9068754/T)*exp(-
392.7811175/T)*exp(-269.3867520/T))) + 1.380000000*10^(-23)*ln(3.631578947*10^(-26)*T) +
2.070000000*10^(-23)*ln(6.568918916*10^18*T) + 3.450000000*10^(-23)) + 3.075035939

S7.3.3: ΔG for rxn(III) from 2 oxygen defect surface

ΔG = -6.24151*10^18*T*(1.380000000*10^(-23)*ln(0.6526881536*sqrt(exp(-2274.585976/T)*T/(1 -
exp(-2274.585976/T)))*(1 - exp(-1373.843483/T))*(1 - exp(-603.9022826/T))*(1 - exp(-
315.7980409/T))/(exp(-1373.843483/T)*exp(-603.9022826/T)*exp(-315.7980409/T))) +
6.900000000*10^(-24)*ln(3.631578947*10^(-26)*T) + 1.035000000*10^(-23)*ln(1.049368400*10^19*T)
+ 1.725000000*10^(-23)) + 3.703462374
